# One‐Size‐Fits‐All: A Universal Binding Site for Single‐Layer Metal Cluster Self‐Assembly

**DOI:** 10.1002/advs.202508034

**Published:** 2025-07-04

**Authors:** Emerson C. Kohlrausch, Sadegh Ghaderzadeh, Gazi N. Aliev, Ilya Popov, Fatmah Saad, Eman Alharbi, Quentin M. Ramasse, Graham A. Rance, Mohsen Danaie, Madasamy Thangamuthu, Mathew Young, Richard Plummer, David J. Morgan, Wolfgang Theis, Elena Besley, Andrei N. Khlobystov, Jesum Alves Fernandes

**Affiliations:** ^1^ School of Chemistry University of Nottingham Nottingham NG7 2RD UK; ^2^ School of Physics & Astronomy University of Birmingham Birmingham B15 2TT UK; ^3^ Department of Physics College of Science Qassim University Buraydah 52571 Saudi Arabia; ^4^ SuperSTEM Laboratory SciTech Daresbury Campus Daresbury WA4 4AD UK; ^5^ School of Chemical Process and Engineering and School of Physics and Astronomy University of Leeds Leeds LS2 9JT UK; ^6^ Nanoscale and Microscale Research Centre University of Nottingham Nottingham NG7 2RD UK; ^7^ Electron Physical Science Imaging Centre (ePSIC) Diamond Light source Didcot OX11 0DE UK; ^8^ Cardiff Catalysis Institute School of Chemistry Cardiff University Park Place Cardiff CF10 3AT UK

**Keywords:** correlated single‐atom, defect engineering, nanocluster, single‐layer metal clusters, single‐atom

## Abstract

2D metal clusters maximize atom–surface interactions, making them highly attractive for energy and electronic technologies. However, their fabrication remains extremely challenging because they are thermodynamically unstable. Current methods are limited to element‐specific binding sites or confinement of metals between layers, with no universal strategy achieved to date. Here, a general approach is presented that uses vacancy defects as universal binding sites to fabricate single‐layer metal clusters (SLMC). It is demonstrated that the density of these vacancies governs metal atom diffusion and bonding to the surface, overriding the metal's physicochemical properties. Crucially, the reactivity of vacancy sites must be preserved prior to metal deposition to enable SLMC formation. This strategy is demonstrated across 21 elements and their mixtures, yielding SLMC with areal densities up to 4.3 atoms∙nm⁻^2^, without heteroatom doping, while maintaining high thermal, environmental, and electrochemical stability. These findings provide a universal strategy for stabilizing SLMC, eliminating the need for element‐specific synthesis and metal confinement protocols and offering a strategy for efficiently utilizing metals.

## Graphical abstract

A universal strategy enables the formation of single‐layer metal clusters (SLMC) across 21 elements by harnessing vacancy defects as binding sites. Crucially, synthesis occurs without reactive species, preventing site passivation and ensuring strong metal–surface bonding. This dopant‐free, confinement‐free method achieves record areal densities and long‐term stability, offering a transformative route to 2D metals for catalysis and quantum applications.

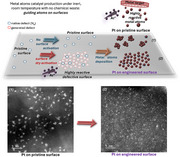

## 1 Introduction

Supported single‐layer metal clusters (SLMC), ranging from single atoms to a few atoms, are crucial for maintaining and advancing sustainable technologies such as energy storage and catalysis, where they outperform traditional multi‐layer clusters and nanoparticles by ensuring that every metal atom in the system is functional.^[^ ^1^ ^]^ Unlike 3D clusters and nanoparticles, SLMC offers a fully exposed metal‐support and metal‐molecule interfaces, simultaneously enhancing interfacial charge transport, consequently improving the tunability of metal electronic properties while ensuring the full availability of metal atoms for catalysis and energy‐related applications.^[^ ^2^ ^]^ However, the highly dynamic and reactive nature of metal atoms on surfaces presents a significant challenge in synthesizing SLMC with high surface coverage and stability, which is usually achieved through confinement between layers of the support, thereby limiting their full potential and applicability.^[^ ^3^ ^]^

An alternative strategy currently employed for SLMC synthesis on carbon materials involves heteroatom doping, such as nitrogen (N),^[^ ^4^ ^]^ fluorine (F),^[^ ^5^ ^]^ and phosphorus (P),^[^ ^6^ ^]^ typically involving complex synthetic steps and high‐temperature treatment.^[^ ^7^ ^]^ Heteroatom doping strengthens metal‐support interactions, stabilizing SLMC and preventing the formation of 3D system.^[^ ^8^ ^]^ For example, when Pt atoms are deposited on nitrogen‐doped carbon, the binding energy increases from –1.6 eV for Pt–C to –2.4 eV for Pt–N, demonstrating the importance of strong metal‐support interactions in stabilizing SLMC (Table S1, Supporting Information). Among potential stabilization strategies, defect creation—such as vacancies in carbon lattices—offers the highest binding energy for stabilizing single metal atoms and thus SLMC.^[^ ^9^ ^]^ In the case of Pt, single vacancies (Pt–Cv bonding = –7.8 eV) enhance the binding energy eightfold compared to Pt–C and threefold compared to Pt–N/C, surpassing all other stabilizing agents (Table S1 and Figure S1, Supporting Information).^[^ ^10^ ^]^ Existing methods for generating defects include ion and electron beam bombardment,^[^ ^11^ ^]^ chemical vapor deposition,^[^ ^12^ ^]^ plasma treatment,^[^ ^13^ ^]^ and thermal and wet chemical etching^[^ ^14^ ^]^ (see details in Table S17, Supporting Information). However, the key challenge is to create such sites under controlled, inert, and mild conditions using a scalable approach, as they are highly reactive and become immediately occupied under ambient conditions.^[^ ^15^ ^]^ Achieving this requires a clean environment to generate vacancies and a finely controlled flow of single metal atoms onto the surface, all within the same inert setting, which has yet to be achieved.

Here, we demonstrate a scalable and universal method to control the dynamic behavior of metal atoms on carbon surfaces, enabling a high density of single‐layer metal clusters. This is achieved through in situ generation of highly active binding sites, followed by a flow of atomically dispersed metal, using argon ions as the only stimulus for both processes. This approach allows precise control over the ratio of binding sites to metal atoms, enabling 98% of deposited Pt to form SLMC. The key aspect of this method is that it occurs in the absence of reactive species, such as air or solvents, preventing the passivation of the binding sites and ensuring effective bonding between metal atoms and vacancies. Using this approach, we achieve the highest reported areal density of Pt SLMC on carbon to date, 4.3 atoms∙nm^−^^2^, without requiring heteroatom doping. Furthermore, the broad applicability of this approach is demonstrated by the synthesis of an SLMC library encompassing 21 different elements, 3 different supports and the successful incorporation of up to 3 distinct metals into SLMC alloys. These materials exhibit exceptional thermal stability at 200 °C and maintain electrochemical stability over 10 h of operation, remaining unchanged after 16 months of air exposure post‐production.

## 2 Results and Discussion

### 2.1 On‐Surface Synthesis of Single‐Layer Metal Clusters (SLMC)

**Figure 1:**
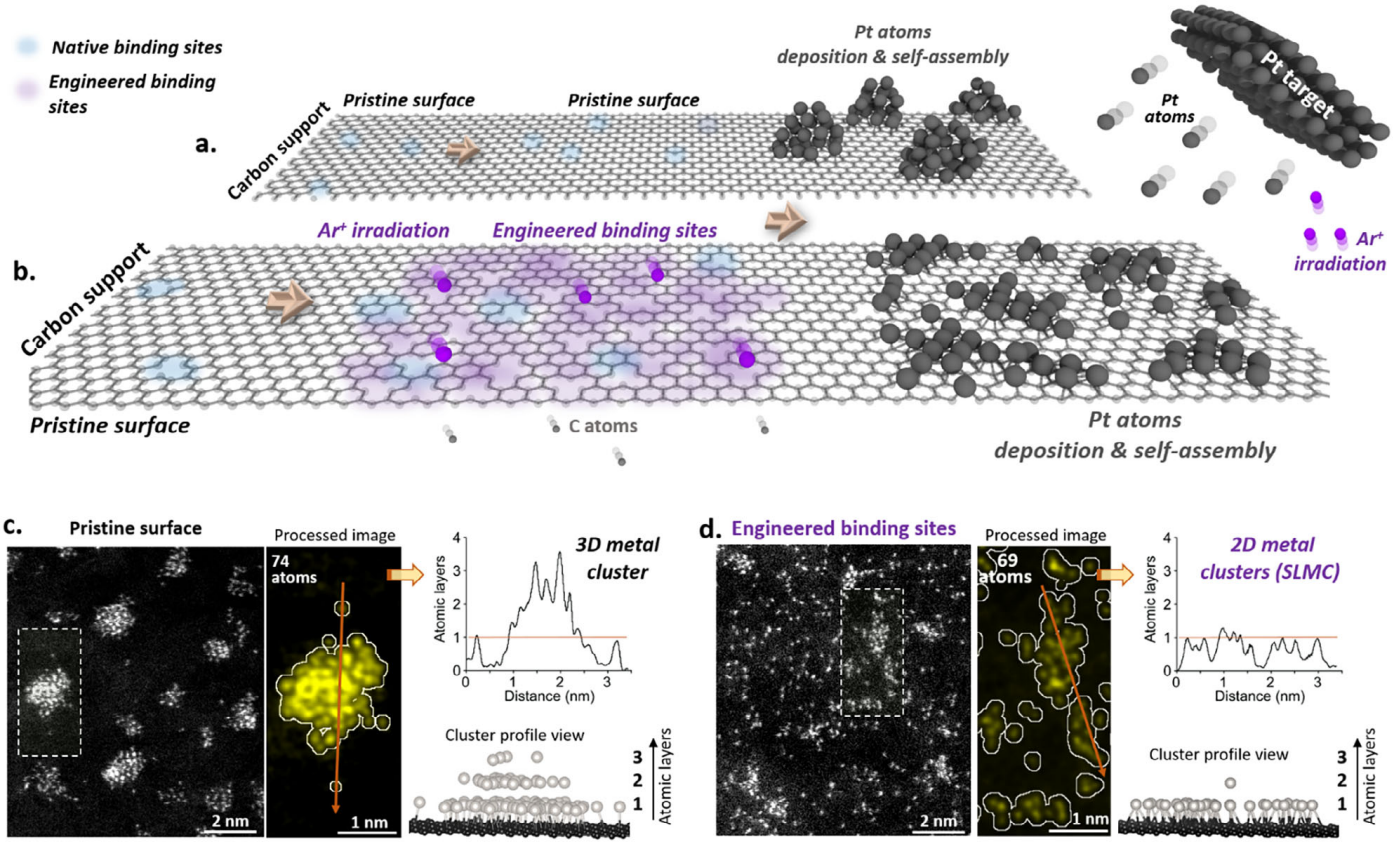
Method for producing high‐coverage single‐layer metal clusters (SLMC) and 3D clusters for comparison. a) Schematic illustration of a pristine carbon support exposed to a flux of Pt atoms, resulting in the formation of 3D clusters at the native binding sites on the carbon surface. b) Argon ion irradiation of the carbon surface generates defect sites, which, upon exposure to Pt atoms, lead to the formation of high‐coverage SLMC stabilized at the engineered defects. c) Pristine surface: AC‐STEM cropped image and corresponding processed image showing a ≈87‐atom 3D Pt cluster formed on a pristine carbon support. The line profile indicates stacking up to three atomic layers. d) Engineered binding sites: AC‐STEM image and corresponding processed image showing a ≈76‐atom SLMC formed on an argon‐irradiated carbon surface. The line profile confirms a monolayer structure. Atomic models (side view) derived from processed images are shown for both (c,d) with the respective AC‐STEM images simulation shown in Figure S11 (Supporting Information).

The bottom‐up assembly of metal clusters typically occurs on support surfaces, with the resulting morphology governed by factors such as metal coverage, temperature, and, critically, the type and concentration of defects within the support material.^[^ ^15a^ ^]^ For instance, when Pt atoms generated via magnetron sputtering (Figure S2, Supporting Information) are deposited onto pristine carbon particles, the formation of 3D clusters is favored (**Figure** **1**a,c). This can be associate to the low concentration of strong binding sites, which results in high mobility of the metal atoms on carbon surfaces. To achieve high‐density formation of SLMC, our strategy centers on modulating atomic diffusion by increasing the density of binding sites, such as vacancy defects, on the support surface prior to metal deposition. Importantly, both steps are conducted under an inert atmosphere to avoid surface passivation. Defect generation is achieved by irradiating the carbon support with argon ions at energies exceeding 41 eV, the calculated displacement threshold for carbon atoms (see Experimental section for details), resulting in a high density of surface defects. These defects act as effective binding sites for incoming metal atoms, restricting their mobility and promoting SLMC formation (Figure 1b,d).

Aberration‐corrected scanning transmission electron microscopy (AC‐STEM) imaging and corresponding line profile analysis can distinguish between 3D clusters and SLMC (Figure 1c,d; Figure S11 Supporting Information). In Figure 1c, metal atoms on the pristine carbon surface preferentially assemble into a 3D cluster with 99% of atoms contributing to vertical growth. In contrast, metal atoms on a surface with a high density of binding sites form exclusively SLMC with 7 ± 4 atoms (Figure 1d and Table S8, Supporting Information). The AC‐STEM image reveals that 97% of atoms are located on the plane of the support, with the line profile and image simulation confirming the monolayer structure. This direct comparison highlights that a higher concentration of strong binding sites, such as defects, suppresses vertical growth and confines metal atoms to a single‐layer morphology.

To further demonstrate the control of metal atom mobility via defect engineering, we systematically varied the concentration of defects on carbon surfaces by adjusting the argon ion irradiation conditions. The defect concentration corresponding to different irradiation times was monitored through changes in the surface fraction of *sp^3^*‐hybridized carbon, which correlates with increasing carbon lattice distortions induced by higher defect concentrations.^[^ ^16^ ^]^ The variation in *sp^3^*‐carbon density, and thus the relative defect concentration, was quantified by measuring the *D*‐parameter, derived from the C KLL Auger transition in X‐ray Photoelectron Spectroscopy (XPS) measurements (Section S4.2, Supporting Information).^[^ ^17^ ^]^

Carbon, in its pristine form without argon ion irradiation (CP), exhibits ≈4% surface of *sp^3^*‐hybridized carbon atoms attributed to native defects. When carbon particle surfaces are irradiated with argon ions for durations ranging from 10 to 60 s, at ion energies between 50 and 100 eV, a linear increase in defect concentration is observed (Table S5, Supporting Information), rising from 4% in the CP to 6% after 5 s (C6%), 16% after 15 s (C16%), and 35% after 60 s (C35%) of argon ion irradiation. The increase in surface defect concentration was also confirmed through changes in the π–π* contributions in the XPS of C 1s (Figure S7, Supporting Information),^[^ ^16^ ^]^ as well as through changes in the Raman spectra (Figures S8 and S9, and Table S6, Supporting Information).^[^ ^18^ ^]^ The effect of different binding site concentrations on SLMC formation was evaluated by submitting all samples to the same Pt loading (4 ± 0.5 atoms∙nm^2^) (Figure 1a and Section S4.3, Supporting Information).

**Figure 2:**
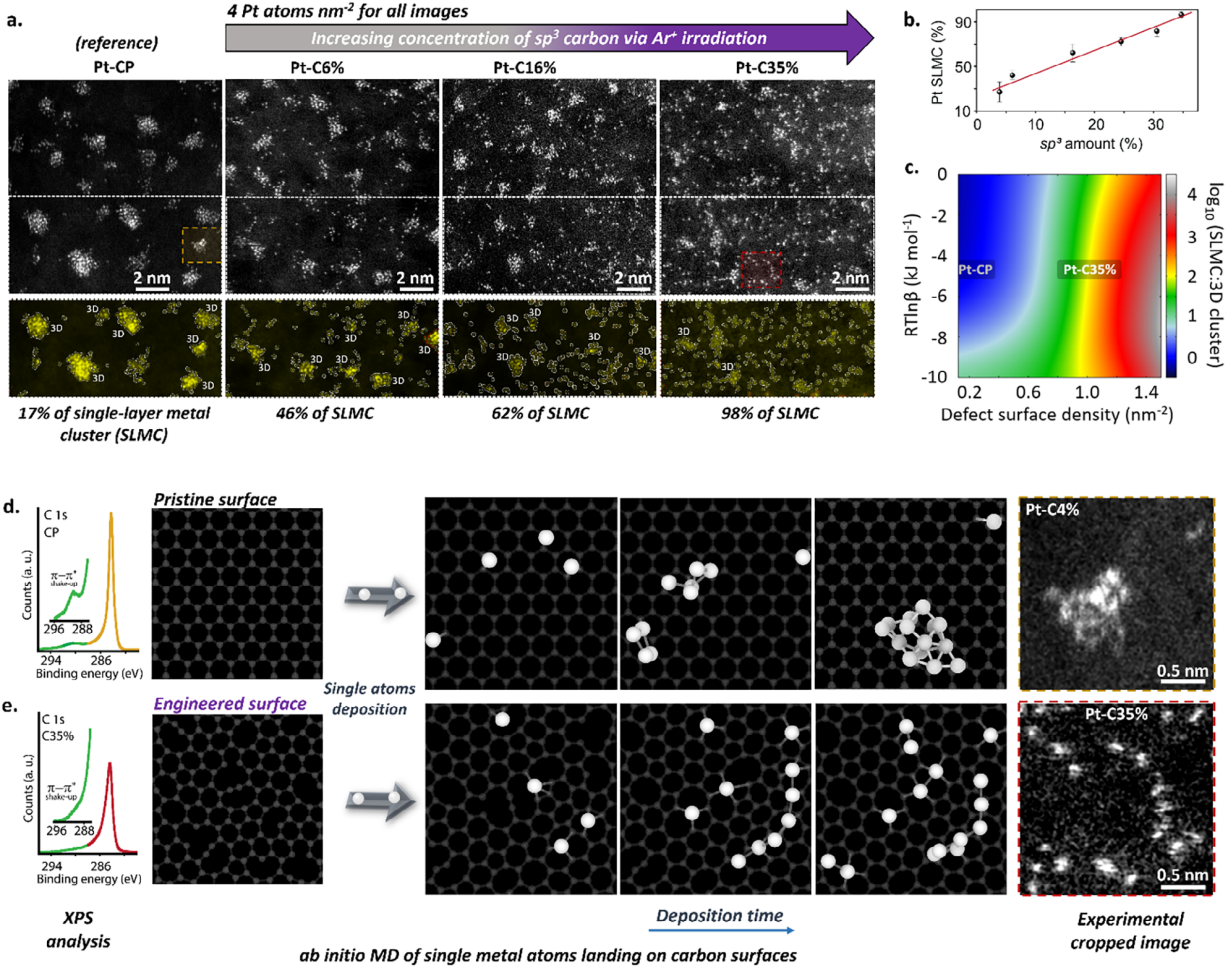
Effect of increasing defect concentration on the carbon surface in the formation of single‐layer metal cluster (SLMC). a) AC‐STEM images revealing the increase in Pt surface atoms on carbon surfaces with increasing Ar ion irradiation exposure. Note that the total Pt loading is maintained constant across all samples. b) Graph illustrating a linear correlation between the *sp^3^* amount and the percentage of SLMC formed on the carbon surface, indicating that increases in *sp^3^* content correspond to higher SLMC formation rates. c) Plot in a parametric space showing a ratio of surface densities of SLMCs and 3D clusters as a function of point defect concentration, *T* ‐ temperature, R ‐ universal gas constant and *β* ‐ barrier for metal atoms joining a cluster. d) XPS C 1s spectra of pristine and engineered carbon surfaces, showing a qualitative low and high defect concentration, respectively, indicated by the presence or absence of the π–π* shake‐up signal. ab initio molecular dynamics (AIMD) simulations demonstrate the formation of 3D clusters on pristine surfaces, whereas SLMC forms on engineered surfaces, exhibiting a striking similarity to experimental results (cropped images on the right). The Pt atoms (large white spheres) are dropped at identical locations on both surfaces.

AC‐STEM images show that Pt 3D clusters are preferentially formed on CP surfaces, with only 19% of the deposited Pt atoms stabilized as SLMC. In contrast, SLMC formation significantly increases with the *sp^3^‐*carbon content, reaching 39% on C6%, 51% on C16%, and an exceptional 98% on C35%, indicating near‐complete stabilization of Pt atoms as monolayer species (**Figure** **2**a,b).

To further analyze the relationship between SLMC and surface defect density, we used the kinetic nucleation theory of metal clusters on surfaces with point defects.^[^ ^15a^ ^]^ (Figure 2c). At a defect density of ≈0.1 nm⁻^2^, 3D clusters dominate, significantly outnumbering SLMC (dark blue zone). As defect density increases to ≈0.60 nm⁻^2^, the fractions of 3D clusters and SLMC become comparable (light blue zone). Beyond 0.75 nm⁻^2^, SLMC dominate, comprising 90% to 99% of the total (blue‐green to yellow‐red zones). These findings indicate a strong dependence of SLMC fraction on surface defect density, aligning with our experimental observations. This strong control enabled us to achieve the highest reported to date areal density of Pt SLMC on undoped carbon of 4.3 ± 0.1 atoms∙nm^‐^^2^ (Table S9, Supporting Information), which is ca. ten times higher than reported that on undoped and N‐doped carbon, the later limited by the N doping density.^[^ ^7^, ^15^ ^]^ To further validate the applicability of this approach for controlling metal diffusion on different surfaces, Pt atoms were deposited on graphitized carbon nanofibers, graphite flakes, and hexagonal boron nitride, yielding similar results (Section S4.4, Supporting Information).

We employ *a* *b* *initio* molecular dynamics (AIMD) modeling to investigate the dynamics of Pt metal atoms deposition on carbon surfaces with varying concentrations of binding sites (Figure 2c,d and Videos S1 and S2, Supporting Information).^[^ ^19^ ^]^ In these simulations, Pt atoms gradually land on the surface, one by one, diffusing on the surface at room temperature, emulating the experimental conditions. On a pristine surface, the adsorption energy of Pt is −1.61 eV, which is markedly lower than the Pt‐Pt binding energy of −3.1 eV (Table S2, Supporting Information). This indicates that Pt‐Pt bonding is thermodynamically more favorable than Pt adsorption on a pristine surface.^[^ ^20^ ^]^ Additionally, Pt atoms on pristine carbon surfaces have low diffusion barriers, resulting in high mobility and easy migration across the surface.^[^ ^21^ ^]^ Consequently, large metal‐free regions persist, promoting Pt 3D cluster formation on CP surfaces (Figure 2b). In contrast, the Pt‐Pt bonding has a lower binding energy than Pt‐Cv on the surface with vacancies, the latter being −7.83 eV (Video S2 and Table S2, Supporting Information). This limits the diffusion of Pt atoms as they become entrapped by vacancy defects, leading to a homogeneous distribution of Pt SLMC across the surface without large areas devoid of the metal, which is in agreement with experimental observations (Figure 2c).

### 2.2 The Critical Role of Defect Oxidation on the Formation of SLMC

The carbon dangling bonds generated by argon ion irradiation are highly reactive and can spontaneously oxidize when exposed to air.^[^ ^15^, ^16^, ^22^ ^]^ This oxidation process is the main hurdle for efficient utilization of vacancies defects for stabilizing SLMC using traditional wet‐chemistry methodologies. To demonstrate that, we conducted both AIMD modeling and controlled experimental depositions, exposing the generated binding sites to ambient conditions prior to metal deposition.

**Figure 3:**
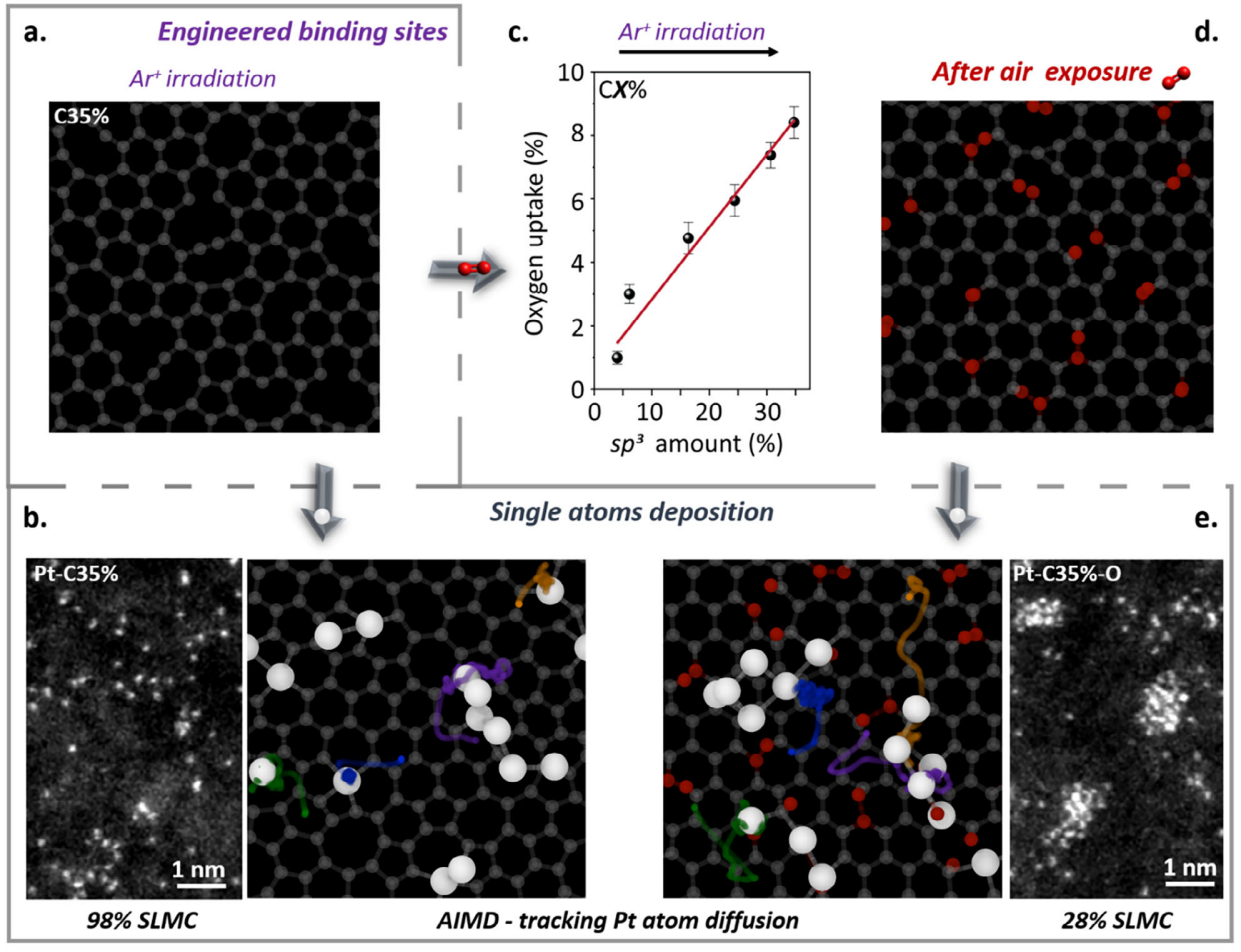
Effect of different environments after defect generation on carbon surfaces. a) AIMD simulation of a carbon surface after argon ion irradiation. b) AIMD simulation of metal atom dynamics on carbon surfaces without air exposure, showing the formation of single‐layer metal cluster (SLMC) as AC‐STEM experimental image on the left. c,d) XPS analysis of the O 1s region of engineered carbon surfaces after air exposure, revealing a linear uptake of oxygen, which is also observed in AIMD simulations (d). e) AIMD simulations of metal atom dynamics on a carbon surface in the presence of oxygen show a significant increase in metal atom diffusion and subsequent clustering, as shown in the AC‐STEM experimental image on the right. The Pt atoms (large white spheres) are dropped at identical locations on both surfaces. The traces of four Pt atoms are shown with different colors to show their trajectory from their landing location to their final position on the surface. The oxygen atoms are shown in red.

Our AIMD calculations show that the vacancies readily bind dioxygen molecules, which then evolve to form C–O functional groups (**Figure** **3**a,d). XPS analysis confirms the presence of oxygen in the carbon support, with the percentage of oxygen rising from 0.7 at.% to 8.4 at.% depending on the surface density of defects, which also correlates linearly with the fraction of *sp^3^*‐C atoms in carbon surface (Figure 3b). Inspecting the structural models predicted by AIMD reveals that the dangling C‐C bonds are saturated, and the the defect is sterically blocked by the oxygen groups, drastically affecting the diffusion dynamics and the chemical interactions of Pt atoms after landing into surface (Figure 3e and Video S3, Supporting Information).^[^ ^15c^ ^]^ For example, a Pt atom (indicated by orange diffusion tracks) diffuses ≈0.9 nm, passing oxidized defects without bonding until it reaches other Pt atoms (Figure 3e). In contrast, in an identical modeling where the defects remain unoxidized, the Pt atom at the same position rapidly bonds with the nearest available defect, exhibiting a significantly shorter diffusion distance of ≈0.2 nm (orange diffusion track; Figure 3b). Remarkably, our experiments, where active binding sites were generated by argon ion irradiation (C35%) and subsequently exposed to ambient air between 5 and 240 min before Pt atoms deposition (Pt‐C35%‐O), confirm the AIMD modeling results. Quantitative analysis of AC‐STEM images from Pt‐C35%‐O, revealed a significant reduction of SLMC upon air exposure. After 5 min of air exposure, the SLMC content decreased sharply to 49%, followed by gradual reductions to 43%, 39%, 29%, and 27% after 30, 60, 120, and 240 min, respectively. This corresponds to a reduction in SLMC formation of ca. 37%, regardless of exposure duration, which is 2.5 times lower than the 98% SLMC formation observed in the same material without air exposure (Figure S15, Supporting Information; Figure 3b,e, respectively). These results demonstrate that the key to success is to ensure that both processes take place in the absence of reactive media, such as air or solvents, to prevent the passivation of both the binding sites and metal atoms, thereby allowing them to bond effectively to each other.

### 2.3 Single‐Layer Metal Cluster Library

**Figure 4:**
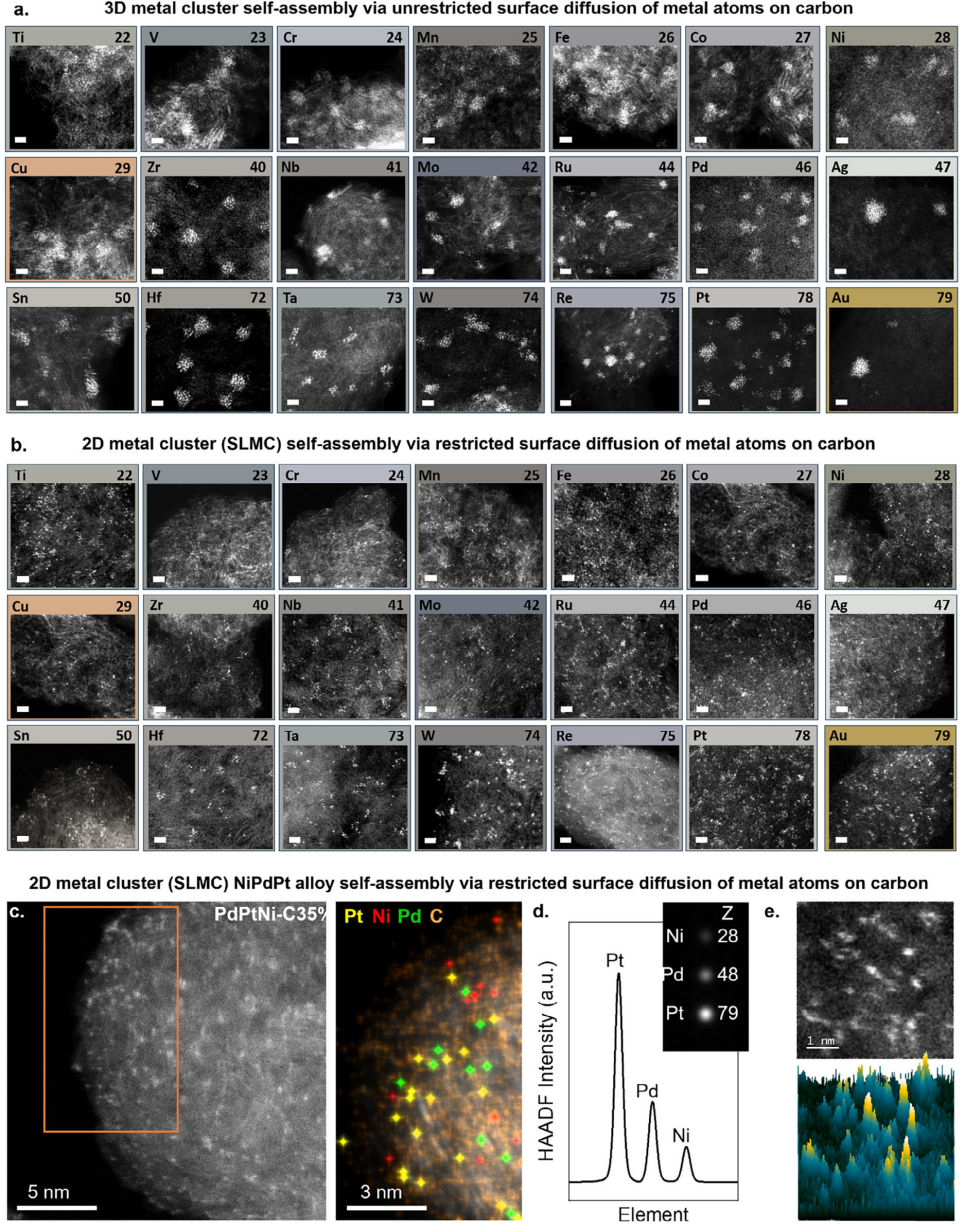
Metal library and multimetallic system reveal binding site density controls 3D vs single‐layer metal cluster (SLMC). a) AC‐STEM images of 21 elements (20 transition metals and a p‐block (Sn)) on pristine carbon surfaces, showing the formation of 3D clusters with large empty regions. b) AC‐STEM images reveal that under a high‐density binding sites, SLMC predominantly form across all the studied elements (scale bar is 1 nm), c) AC‐STEM of a NiPdPt on a C35% showing the completely metal dispersion (right), Single‐atom EDX confirms the presence of Pt, Ni, and Pd, showing a random alloy distribution on the left (Figure S45, Supporting Information). d) Simulated HAADF‐STEM intensity profile of Pt, Pd, and Ni showing the Z number intensity dependence. The inset shows the simulated HAADF‐STEM image for the respective atom. e) Cropped box region from NiPdPt‐C35% samples (top) with the respective intensity surface plot (bottom) showing a qualitative distribution of the trimetallic system across the sample surface.

A key challenge in designing universal binding sites lies in the wide variation of physicochemical properties across the periodic table.^[^ ^23^ ^]^ To demonstrate the generality of our approach, we have performed experiments with 20 transition metals and one p‐block element (Sn) using C35% and CP for comparison (**Figure** **4** and Table S13 and Section S4.6, Supporting Information). AC‐STEM imaging revealed a high fraction of SLMC on C35% across all tested elements, with SLMC yields ranging from 71% to 100%. In contrast, elements deposited onto CP primarily formed 3D clusters, with SLMC fractions between 3% and 33%. To rationalize these results, we performed density functional theory (DFT) calculations revealing a wide variation in metal adsorption energies to carbon surfaces, ranging from 0.3 eV for Ag to 2.6 eV for W, as well as distinct metal‐metal bonding strengths atop graphene, spanning 0.9 eV for Cr to 7.7 eV for W (Figure S1 and Table S2, Supporting Information). A general trend emerges across the d‐block, as we move from Group 4 to Group 11, metal‐vacancy bonding becomes increasingly comparable to metal‐metal bonding, correlating with progressive filling of the d‐orbitals.

However, once a critical *sp^3^*‐carbon content is reached, this trend is entirely disrupted, as the generated defects bind strongly, independent of the metal's physicochemical properties, effectively driving the formation and stabilization of SLMC. Even for Group 11 elements such as Au and Ag, where the energy difference between binding modes is minimal, our experiments confirm that SLMC dispersion is still achieved on C35%, increasing from 3% on CP to 90% on C35%. In such cases, kinetic factors become as important as thermodynamics. The ratio between SLMC and 3D clusters is governed not only by the energy balance between metal–metal and metal–vacancy interactions but also by the surface concentration of defects. Early in the deposition process, vacancy sites outnumber metal atoms, making them more likely to bind to vacancies than to each other, even when the energy difference between the two interactions is minimal, as observed for Ag and Au. As a result, high SLMC concentrations are still preferentially achieved when the surface defect density exceeds that of the deposited metal during the initial stages of metal atom deposition. Additionally, we probe the chemical identity of the metal atoms on C35% via XPS. A general trend is that in SLMC the metals on the left of the d‐block assume higher oxidation states than on the right side, reflecting the periodicity of d‐orbital filling in transition metals (Table S14 and Figures S36–S38, Supporting Information).^[^ ^24^ ^]^

### 2.4 Multimetallic Single‐Layer Metal Cluster

Beyond monometallic systems, we demonstrated that our approach effectively stabilizes SLMC mixtures of elements (Figure 4c–e; Figures S39–S43 and Section S4.7, Supporting Information). Since different metals exhibit varying binding affinities to carbon species and each other, competitive binding at vacancy sites could potentially lead to preferential adsorption of one metal over another, promoting clustering.^[^ ^25^ ^]^ To investigate this, we selected Ni, Pd, and Pt from Group 10 of the transition metals, as Ni and Pt share similar binding characteristics to carbon, whereas Pd exhibits lower binding energy (Table S2, Supporting Information), providing a basis for comparing metal competition at vacancy sites versus 3D cluster formation. These metals were co‐deposited onto CP and C35% supports to form bimetallic (NiPd, NiPt, PdPt) and trimetallic (NiPdPt) systems (Section S4.7, Supporting Information). AC‐STEM imaging revealed a high fraction of SLMC on C35%, with NiPd and NiPt reaching 98% SLMC and PdPt 96%, contrasting with their counterparts deposited onto CP with 30%, 26% and 23% of SLMC formation, respectively. A similar result was achieved for the trimetallic NiPdPt system with 40% on CP to 100% of SLMC on C35% (Figure 4a,b). Atomic‐resolution EDX/EELS mapping, combined with single‐atom contrast analysis, confirmed that Ni, Pd, and Pt are dispersed randomly on the C35% surface (Figure 4c,e; Figure S44 and Video S4, Supporting Information).^[^ ^26^ ^]^ Additionally, we tested the thermal stability of SLMC NiPdPt‐C35% by heating in an argon atmosphere for 2 h at 200 °C, and environmental stability after a prolonged exposure to air. Remarkably, all metals remained atomically dispersed after even 16 months post‐synthesis, underscoring the robustness of our SLMC stabilization strategy for both mono‐ and multi‐metallic SLMC materials (Figure S45, Supporting Information). Electrochemical stability was further demonstrated by Pt–C35%, which maintained a steady current density over 10 h of continuous operation in 0.5 M H₂SO₄ at 0.16 V vs RHE (Figure S47, Supporting Information).

## 3 Conclusion

In summary, we report the formation of universal binding sites that effectively bind 21 chemical elements, achieving a record of areal density of single‐layer metal clusters (SLMC) at 4.3 atoms nm^−^^2^, more than double the previous record. Our comprehensive experimental and theoretical investigation examines the behavior of various metals on pristine and defect‐engineered supports, including oxidation states, effectively addressing the limitations of earlier wet‐chemistry approaches that obscured true metal‐support interactions. We have identified trends across the d‐block of the periodic table: as we proceed from Group 4 to Group 11, the bonding between metal and vacancies increasingly resembles that of metal‐metal bonding, which promotes 3D cluster formation. However, this trend shifts at critical *sp^3^*‐carbon content, with surface atom diffusion then governed by vacancy defects, independent of the metal's properties, thereby enabling the formation of SLMC. Theoretical models have predicted these dynamics, and our approach successfully translates those predictions into scalable material fabrication. The resulting SLMC materials are exceptionally stable, enduring temperatures of at least 200 °C and maintaining integrity in ambient environments for at least 16 months, as well as under electrochemical operation for at least 10 h. These findings open new avenues for fabricate SLMC, establishing defect engineering as a key principle in the fabrication of 2D metal materials enabling areal densities previously considered inaccessible. This approach holds potential for a wide range of applications in catalysis, energy conversion, and quantum technologies.

## 4 Experimental Section

### Sample Preparation

All the support materials were purchased from Sigma Aldrich, while the metal targets were purchased from Kurt J. Lesker and AJA Internation, and used without further treatment. The supports include Vulcan XC 72R, labeled as CP for pristine material and CX% for the samples that have undergone argon ion irradiation, where X represents the percentage of *sp^3^* hybridization determined by XPS analysis. Other supports utilized in this study include graphitized nanofibers (GNF), graphite flakes (GF), and hexagonal boron nitride (h‐BN). The powder supports were dispersed in isopropyl alcohol, sonicated for 15 min, then drop‐cast onto a holey carbon TEM grid and carbon paper, and then left to air dry for at least 2 h prior to argon irradiation or metal deposition. Depending on the deposited metal, copper, nickel, and gold mesh holey carbon film TEM grids (Agar Scientific, UK) were used. The powder surface treatment and metal atom deposition onto surface‐treated powders were performed using a custom‐designed AJA International magnetron sputtering system (Figure S4, Supporting Information). High‐purity argon gas (99.999%) was used for both surface treatment and metal deposition. For the surface treatment, argon ion irradiation was used to promote the formation of defects on the surface of the material, where a range of pressure and power was used to optimize the defect density. The metal atom deposition was conducted under a controlled working pressure. A range of argon pressures and power settings were applied to obtain a total metal loading of 4.0 ± 0.5 atoms per nm^2^. High‐purity argon gas (99.999%) was used for both surface treatment and metal deposition (see Supporting Information for more details). The metal targets used in the magnetron sputtering process included: Ti (99.99%), V (99.99%), Cr (99.99%), Mn (99.99%), Fe (99.99%), Co (99.99%), Ni (99.99%)%), Cu (99.95%), Zr (99.99%), Nb (99.99%), Mo (99.99%), Ru (99.99%), Pd (99.99%), Ag (99.99%), Sn (99.99%), Hf (99.95%), Ta (99.99%), W (99.99%), Re (99.99%), Pt (99.99%), and Au (99.99%).

### Electron Microscopy Measurements

Aberration‐corrected STEM (AC‐STEM) measurements were performed using a JEM‐2100F TEM (JEOL, Japan) operated at 200 kV. The instrument was equipped with a spherical aberration (Cs) probe corrector for STEM (CEOS, Germany). The beam convergence half‐angle was 19 mrad, and the annular dark field (ADF) detector had a collection half‐angle range of 31–82 mrad. AC‐STEM images were acquired with a scanning area of 1024 × 1024 pixels. A bright field (BF) detector was also used in parallel. The single‐atom dispersion for the multimetallic system was investigated at atomic resolution in an aberration‐corrected Nion UltraSTEM100 dedicated Scanning Transmission Electron Microscope (STEM) operated at 60 kV, at the SuperSTEM laboratory, Daresbury, UK. The instrument was equipped with a cold field emission source with a nominal energy spread of 0.3 eV. STEM High‐angle annular dark field (HAADF, with a semi‐angle collection range of 85–180 mrad) images were acquired by rastering a 1 Å corrected probe with a beam convergence half‐angle of 30 mrad and a probe current of ≈25 pA. The EELS data were acquired with a collection half‐angle of 44 mrad using a Gatan Enfina EEL spectrometer retro‐fitted with a MerlinEM direct electron detector optimized for EELS acquisition at low acceleration voltages. A dispersion of 1.16 eV/channel was used to capture a wide range of energy losses. The energy‐dispersive X‐ray spectroscopy (EDXS) data were acquired using a Bruker XFlash 6|100 UHV‐compatible, windowless silicon drift detector. The geometric solid angle of the detector was estimated to be 0.7 sr. Spectrum images and single continuous spectra were acquired using Bruker Esprit 2.2.1 software, requiring minimal raw data processing or background removal due to the very low count‐levels from single‐atom systems. The map in Figure 4c was generated by integration of the raw counts from the Pt Mα, Pd Mα and Ni Kα lines, followed by a 5‐neighbour‐pixel Gaussian smoothing. System counts (in particular from the Cu Lα line due to the support grid materials, and Si Kα, due to ubiquitous Si‐based contaminants in carbon supports) also contributed to the total acquired signal but were not mapped. The additional small area tracking data presented in the Supporting Information was acquired by defining a small scanning window over a cluster of atoms pre‐identified from mapping, which was scanned continuously during 5 min while accumulating both EDXS and EELS spectral data simultaneously, as described previously.^[^ ^26a^ ^]^ The resulting average spectrum confirms the identity of the metal atoms in that specific cluster as Pt and Pd (clear from the EDXS data) and Ni (very faintly visible in the EDXS and difficult to distinguish from the neighboring Cu Lα line but more clearly visible above background in the EELS as an L_2,3_ edge). The series of frames provides an indication of the high dynamic behavior of the atoms under the electron beam. Complementary STEM‐annular dark field (ADF) image simulations were conducted to analyze the brightness contrast of Ni, Pd, and Pt atoms. The simulations were performed using the Atomic Simulation Environment (ASE) and the abTEM library within a Jupyter Notebook platform.^[^ ^27^ ^]^

### Image Analysis Methodology

Image analysis was conducted using a custom‐developed, GUI‐based Python program designed by the research team to identify individual single‐atom species, nanoclusters, and nanoparticles. A detailed description of the analytical process employed in this study is provided below.

### Image Analysis Methodology—Pre‐Processing of Raw Images

The raw images were pre‐processed to remove background noise and irrelevant features using filtering techniques, including Notch and Gaussian filters. These filters smooth the image and enhance the visibility of atomic structures. As a result, only significant features—such as individual atoms or clusters—are retained, ensuring clearer identification.

### Image Analysis Methodology—Detection of Individual Atoms

To identify individual atoms, the Python code detects local maxima, which correspond to bright points in the image, representing atomic centers. A background subtraction and Gaussian filter were applied to further enhance these points, followed by a thresholding step to remove regions with a brightness level below a predefined threshold. This ensures that only potential atoms are highlighted. Local maxima are then identified by comparing each pixel's intensity to its surrounding neighbors.

### Image Analysis Methodology—Identification of Atomic Clusters

Clusters, which consist of multiple atoms grouped together, were detected by applying a circular maximum filter of radius 0.15 nm to the thresholded image. Each connected area represents a cluster. For each cluster, the footprint (the area occupied by the cluster) was extracted, along with the total number of atoms within the cluster. An example of the final image analysis is presented in Figure S3a,b (Supporting Information), where individual atoms, dimers, trimers, and nanoclusters were highlighted.

### Image Analysis Methodology—Definition of Single‐Layer Metal Cluster Percentage and Areal Density

To classify clusters, they were distinguished into two categories: 2D and 3D nanoclusters (Figure S3, Supporting Information). This classification was determined using a 3D thresholding calculation, which assesses whether a cluster was confined to a single layer or extends across multiple layers. The classification process involves dividing the total number of atoms in a cluster by the number of atoms in a single atomic layer, using the footprint. If the result indicates that the cluster was limited to a single atomic layer, it was classified as 2D (Figure S3c, Supporting Information).^[^ ^28^ ^]^ Conversely, if the cluster extends across multiple layers, it was classified as a 3D cluster (Figure S3c, Supporting Information). Further, the atoms were classified into two categories: i) the single‐layer metal clusters (SLMC), which include isolated atoms, dimers, trimers, and 2D clusters (planar assemblies of four or more atoms forming a monolayer on the support), and ii) 3D clusters, defined as multilayered, volumetric structures. The percentage of atoms in SLMC form was determined by comparing the total number of atoms assigned to the SLMC system against the total number of metal atoms observed in the image. Furthermore, the SLMC areal density (atoms nm^−^^2^) was determined by dividing the total number of atoms within the SLMC system by the area of the support shown in the image.^[^ ^7a^ ^]^

### Image Analysis Methodology—Atomic Model Construction from STEM Images Validation

Following the image analysis and classification of atomic features, representative 3D atomic models were constructed directly from the processed AC‐STEM images to visualize the spatial configuration and layer distribution of the Pt clusters. These models were intended to aid in the interpretation of 2D contrast features, support theoretical simulations, and validate structural assignments. The construction process begins by mapping the 2D coordinates of each detected atomic center—identified through local maxima detection—onto a real‐space coordinate system. The relative brightness of each atomic site, which correlates with projected atomic column height, was then used to estimate the number of atomic layers present at each position. This brightness‐to‐height mapping was calibrated using a normalized intensity scale and discretized to one, two, or three atomic layers, depending on the image conditions and intensity distribution. For each atomic site, accordingly, a number of Pt atoms were assigned in the vertical direction based on the intensity of the local maxima position. This yields a 3D cluster model consistent with the experimentally observed morphology. Furthermore, the generated atomic model were use to simulate an AC‐STEM image using the Atomic Simulation Environment (ASE) and the abTEM library within a Jupyter Notebook platform. The simulated data includes shot noise and random horizontal displacements of the individual scan lines. The magnitudes of both were chosen to roughly reflect the corresponding noise in the experimental data.^[^ ^27^ ^]^

### X‐Ray Photoelectron Spectroscopy (XPS) Measurements

XPS measurements were performed using a Thermo Scientific K‐Alpha spectrometer equipped with a monochromatic Al Kα radiation source operating at 72 W (6 mA × 12 kV). The analysis area was ≈400 × 600 microns, and all experimental conditions are detailed in Table S3 (Supporting Information). All samples were analyzed using a dual ion‐electron charge compenzation detector, operating at an argon background pressure of 10⁻⁷ mbar. Samples were mounted by pressing them onto silicone‐free, double‐sided adhesive tape. Data processing was conducted using CASAXPS (Version 2.3.27), with charge correction applied to the graphitic C 1s peak of 284.5 eV.

### Electrocatalysis

All measurements were performed using a conventional three‐electrode system. This setup included a Pt‐C35% acting as a working electrode, a Hg/HgSO_4_ reference electrode, and a graphite rod counter electrode, with measurements taken using an electrochemical analyzer (IVIUM Technologies). Linear sweep voltammetry and chronoamperometry measurements were performed in 0.5 M H_2_SO_4_ electrolyte. The observed potentials against the Hg/HgSO_4_ reference were converted to the RHE scale using the Nernst equation: *E_(RHE)_* = *E_(Hg/HgSO4)_* + 0.68 + 0.0596 × pH. The electrochemical cell was purged with argon for 15 min prior to the measurements to eliminate residual oxygen.

### Computational Details

The ab‐initio molecular dynamics (AIMD) calculations were performed using the CP2K package based on PBE functional and a hybrid Gaussian/Plane‐Wave scheme (GPW).^[^ ^19^ ^]^ The valence electrons were expanded in Double‐Gaussian basis sets with one polarization function (DZVP) optimized for multi‐grid integration, while the core electrons and nuclei were described by Goedecker–Teter–Hutter (GTH) pseudopotentials.^[^ ^19^, ^29^ ^]^ Four multi‐grids and a cutoff of 300 Ry were used in this study. Van der Waals corrections were accounted for using the DFT‐D3 method of Grimme.^[^ ^30^ ^]^ The simulation cell contained 160 carbon atoms in the pristine graphene system. The geometry of all the structures was carefully optimized.

The displacement energy threshold of carbon atoms in graphene under argon and Pt ion irradiation was calculated within the NVE ensemble.^[^ ^31^ ^]^ The AIMD calculations for the metal depositions were performed within the NVT ensemble at 300 K. Three distinct graphitic environments were considered: pristine, Ar‐treated, and Ar‐treated followed by air exposure. All graphene structures were annealed at 300 K to reach equilibrium before metal deposition. Pt atoms, at a loading consistent with experimental conditions (4 atoms nm^−^^2^), were deposited one at a time with a kinetic energy of 8 eV at identical locations across the three environments. After each deposition, the system was equilibrated for ≈1 ps before the next atom was added. Following the deposition of all metal atoms, the system was maintained at the same temperature until no further changes were observed, ensuring an equilibrium state.

While Pt atoms on pristine graphene sheets exhibited high mobility and tended to form 3D clusters, defective graphene—characterized by monovacancies and sparse divacancies from Ar treatment—restricted Pt atom diffusion. This resulted in metal atoms being irreversibly trapped in single vacancies near their landing sites. The divacancies reconstructed into 5‐8‐5 rings, becoming more stable and less reactive than single vacancies. Pt atoms on this surface showed significantly enhanced dispersion compared to the pristine system with identical landing locations. To model air exposure, the same defective graphene from the previous step was used and placed O₂ molecules on the vacancy sites. Remarkably, in multiple instances, Pt atoms landed near defect sites but did not bind to them; instead, they navigated the surface to join other Pt atoms. This indicated that oxygen atoms blocked the vacancies, rendering them inactive for metal‐defect binding, which aligns with the experimental observations of air exposure following Ar treatment. Thus, avoiding air exposure was crucial for fabricating single‐atom and small metal‐cluster formations on graphitic surfaces. Graphene was chosen for AIMD simulations due to its structurally uniform framework, enabling controlled modeling of metal–carbon interactions. Unlike the complex surface of carbon black, graphene allows reliable atomic‐scale simulations. Additionally, the binding energy between Pt atoms and carbon surfaces was not expected to differ significantly between these two materials, as demonstrated by similarity between the AIMD and experimental results in Figure 2d,e.

Spin‐polarized Density Functional Theory (DFT) calculations of binding energies of metal atoms to graphene were performed with the Vienna Ab initio Simulation Package (VASP), within the plane‐wave projector augmented‐wave (PAW) method. The structures were relaxed using the Perdew–Burke–Ernzerhof (PBE) exchange–correlation functional with a force tolerance of 0.005 eV A−1 and an electronic convergence criteria of 10−6 eV.^[^ ^19^, ^32^ ^]^ The energy cut‐off was set to 550 eV, and a gamma point‐centered Monkhorst‐Pack k‐point grid of 5 × 5 × 1 was used to sample the Brillouin zone. Van der Waals interactions were taken into account using the DFT‐D3 method with Becke–Jonson damping function. The pristine graphene supercell contained 98 carbon atoms.^[^ ^30^ ^]^

The energies were calculated as follows:

(1) $$E_{binding}=\left(E_{graphene+metal}-E_{graphene}-E_{metal}\right)$$

where E_graphene+metal_ is the total energy of the metal atom on the graphene sheet, E_graphene_ is the total energy of only the graphene sheet, and Emetal was the total energy of the isolated metal atom in the gas phase.

The diagram in Figure 2c was obtained by solving a system of kinetic equations given in at every point of a 21 × 21 square grid and interpolating the data using the 3D Gaussian function.^[^ ^15a^ ^]^ The flow of atoms to the surface was 4.0 (atoms nm^−2^ s^−1^) and the diffusion coefficient was 7.7 × 10^−14^ (m^2^ s^−1^) which corresponds to the diffusion barrier of 0.36 eV at room temperature. All other parameters of the kinetic model were the same as in. The ratio between SLMC and 3D clusters was calculated as

(2) $$SLMC:3D=\left(\sum_{i=1}^{N}f_{i}\right):\left(\sum_{i=N+1}^{\infty }f_{i}\right)$$

where *f_i_* is the total surface concentration of clusters having i metal atoms as determined from the solution of the kinetic equations. The threshold for distinguishing SLMC from 3D clusters was taken to be *N = 8*.

## Conflict of Interest

The authors declare no conflict of interest.

## Author Contributions

E.C.K. performed the ion irradiation, metal atoms deposition, and part of the characterization, as well as analyzed all data in the manuscript. S.G., I.P., and E.B. were responsible for the theoretical simulations. G.A., F.S., E.A., and W.T. performed AC‐STEM and analyzed the images. G.A.R. performed and analyzed the Raman spectra. Q.M.R. performed single‐atom EDX and spectroscopy. M.T., M.Y., and R.P. carried out part of metal depositions and their characterization. M.D. carried out the ADF intensity simulations. D.M. carried out XPS analysis. A.N.K. and J.A.F. provided funding acquisition and project supervision. The work was written and edited by all co‐authors.

## Supporting information

Supporting Information

Supplemental Video 1

Supplemental Video 2

Supplemental Video 3

Supplemental Video 4

## Data Availability

The data that support the findings of this study are available in the supplementary material of this article.
