# Supplementary material for: One‐Size‐Fits‐All: A Universal Binding Site for Single‐Layer Metal Cluster Self‐Assembly
Source: Adv Sci (Weinh). 2025 Jul 4;12(37):e08034. doi: 10.1002/advs.202508034 (PMC12499407; doi:10.1002/advs.202508034)
Supplement: Supplementary file 1 — Supporting Information [file ADVS-12-e08034-s001.docx]

SUPPORT INFORMATION

**One-size-fits-all: a universal binding site for single-layer metal cluster self-assembly**

Emerson C. Kohlrausch,^1^ Sadegh Ghaderzadeh,^1^ Gazi N. Aliev,^2^ Ilya Popov,^1^ Fatmah Saad,^2^ Eman Alharbi,^2,3^ Quentin M. Ramasse,^4,5^ Graham A. Rance,^6^ Mohsen Danaie,^7^ Madasamy Thangamuthu,^1^ Mathew Young,^1^ Richard Plummer,^1^ David J. Morgan,^8^ Wolfgang Theis,^2^ Elena Besley,^1^ Andrei N. Khlobystov,^1^ Jesum Alves Fernandes^1*^

^1^ School of Chemistry, University of Nottingham, Nottingham, UK, NG7 2RD

^2^ School of Physics & Astronomy, University of Birmingham, Birmingham, UK, B15 2TT

^3^ Department of Physics, College of Science, Qassim University, Buraydah 52571, Saudi Arabia.

^4^ SuperSTEM Laboratory, SciTech Daresbury Campus, Daresbury, UK, WA4 4AD

^5^ School of Chemical Process and Engineering and School of Physics and Astronomy, University of Leeds, Leeds, UK, LS2 9JT

^6^ Nanoscale and Microscale Research Centre, University of Nottingham, Nottingham, UK, NG7 2RD

^7^ Electron Physical Science Imaging Centre (ePSIC), Diamond Light source, Didcot, UK, OX11 0DE

^8^ Cardiff Catalysis Institute, School of Chemistry, Cardiff University, Cardiff, UK, CF10 3AT

**This PDF file includes:**

Captions for Supporting Information Videos S1-S4

Table S1-S17

Figures S1-S47

Supporting Information Text

**1. Captions Supporting Information Videos**

**Supporting Information Video S1** | *Ab-initio* molecular dynamics simulation of 15 atoms Pt landing, one by one, onto 2.7 x 2.7 nm non-defective graphene surface. The video shows the relatively large diffusion of Pt atoms across the carbon surfaces, leading to the formation of 3D Pt cluster.

**Supporting Information Video S2** | *Ab-initio* molecular dynamics simulation of 15 atoms Pt landing, one by one, onto 2.7 x 2.7 nm defective graphene surface. The video shows the relatively short-range diffusion of Pt atoms on the carbon surface, resulting in the formation of a single-layer Pt cluster.

**Supporting Information Video S3** | *Ab initio* molecular dynamics simulation of a 2.7 × 2.7 nm defective graphene surface exposed to an oxygen environment, followed by the sequential 15 atoms Pt landing, one by one, at the sample location used for the modelling presented at supplementary video 2. The video highlights the relatively large diffusion of Pt atoms on the carbon-oxygen surface, ultimately leading to the formation of a Pt 3D cluster similar to that observed on non-defective graphene.

**Supporting Information Video S4** | The series of frames for the square region of the image on Supplementary Figure 45, provides an indication of the high dynamic behaviour of the atoms under the electron beam at 60 KeV.

**2. Computational details**

**Table S1:** Binding energies between metal atoms onto different surface environments.

| Surfaces | Metal-surface | Binding energy (eV) |
| --- | --- | --- |
| Pristine graphene | Pt-C | -1.6 |
| Nitrogen-doped graphene (N-C) | Pt-N-C | -2.4 |
| Graphene with vacancies (C_v_) | Pt-C_v_ | -7.8 |

**Figure S1** | **Metal binding energies on graphene**: adatom adsorption on pristine graphene (E_ads_), metal–metal binding energy of a metal dimer on graphene (E_Metal–Metal_), and binding energy of a metal atom at a carbon single-vacancy (E_Metal–Cv_).


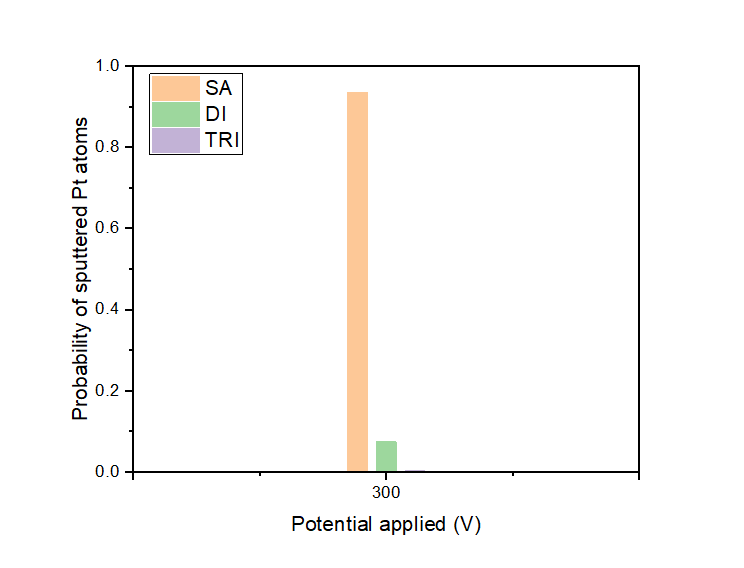


**Figure S2** | Simulated probability distribution of the number of Pt atoms sputtered at the applied potential.

**Table S2:** Adsorption energy (E_ads_) of transition metal atoms on graphene, metal-metal binding energy of 2 atoms on a graphene (M-M) and binding energy of metal on a graphene single carbon vacancy (M-Cv).

| Metal | Adatom on Graphene (E_ads_) | Metal-Metal on Graphene (E_M-M_) | Metal on single carbon vacancy (E_M-Cv_) |
| --- | --- | --- | --- |
| Ti | -1.46415 | -3.59656 | -8.79385 |
| V | -1.08787 | -2.31876 | -7.91192 |
| Cr | -0.54162 | -0.86745 | -6.77228 |
| Mn | -0.33375 | -1.63224 | -6.75044 |
| Fe | -0.57535 | -3.31658 | -7.86635 |
| Co | -1.8331 | -4.79083 | -9.28247 |
| Ni | -1.76968 | -3.07054 | -7.64856 |
| Cu | -0.46185 | -2.68708 | -4.04353 |
| Zr | -1.9416 | -5.72758 | -9.49504 |
| Mo | -0.43253 | -4.18293 | -8.01412 |
| Ru | -1.70102 | -4.71817 | -9.98024 |
| Pd | -1.33745 | -1.943 | -5.91526 |
| Ag | -0.27085 | -1.92879 | -2.14004 |
| Hf | -1.58913 | -5.39545 | -9.56123 |
| Ta | -1.32124 | -5.90257 | -9.92384 |
| W | -2.60257 | -7.22782 | -11.1566 |
| Re | -0.21681 | -5.56215 | -8.80339 |
| Pt | -1.61119 | -3.10244 | -7.83176 |
| Au | -0.40031 | -2.43087 | -2.92864 |

**3. General characterisation**

**3.1 X-ray photoelectron spectroscopy (XPS) measurements**

**Table S3:** Experimental parameters used for the XPS measurements.

| Region | Pass Energy (eV) | Dwell time (s) | Number of scans | Step energy (eV) |
| --- | --- | --- | --- | --- |
| Survey | 160 | 150 | 2 | 0.2 |
| C 1s | 20 | 60 | 20 | 0.05 |
| O 1s | 20 | 60 | 20 | 0.05 |
| C KLL | 100 | 150 | 100 | 0.5 |
| Transitions metals | 20 | 60 | 20-50* | 0.1 |

*Enough scans to obtain a reasonable signal to noise ratio.

**3.2 Micro Raman spectroscopy measurements**

Raman spectroscopy was performed using a HORIBA LabRAM HR Raman spectrometer. Spectra were collected with a 532 nm laser (1%, ~0.3 mW power), a 100× objective, and a 200 µm confocal pinhole. A 600 lines mm⁻¹ rotatable diffraction grating with an 800 mm path length was employed to scan a range of Raman shifts. Spectra were acquired using a Synapse CCD detector (1024 pixels), thermoelectrically cooled to -60 °C. Before spectral acquisition, the instrument was calibrated using the zero-order line and a standard Si(100) reference band at 520.7 cm⁻¹. The spectral resolution in this configuration was better than 1.8 cm⁻¹. To assess structural heterogeneity, spectra were collected from three random locations.

**3.3 Image Analysis Methodology**


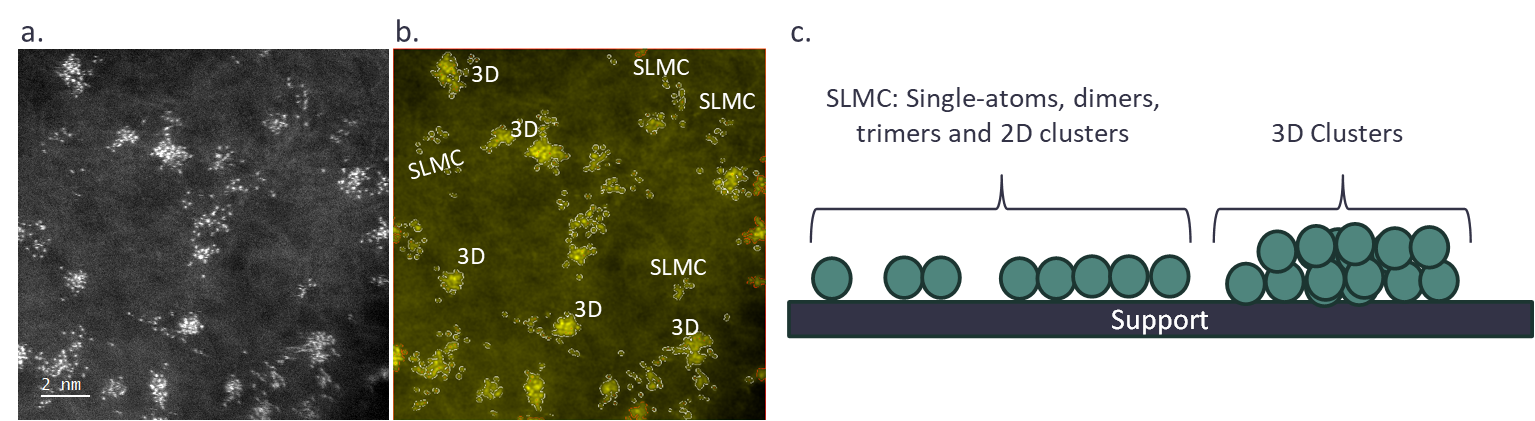


**Figure S3** | (a) AC-STEM raw image of Pt atoms deposited on a Vulcan XC 72R support. (b) Processed version of image (a) after applying the procedure described in the Image Analysis Methodology section. It shows examples of SLMC and 3D Pt clusters. (c) Schematic illustration demonstrating the distinction between SLMC and 3D metal clusters.

**4. Metal atoms deposition onto *in-situ* surface-treated powder support materials via magnetron sputtering**

***4.1 Metal atoms depositions onto surfaces via magnetron sputtering concepts***

The magnetron sputtering process involves the elastic collision of argon ions with a highly pure metallic target, leading to the ejection of atoms or clusters, which are subsequently deposited onto a support material. Our theoretical and experimental studies, conducted over a range of applied potentials up to 500 V, demonstrate that the majority of species ejected from the target are single metal atoms, exceeding 97%. Additionally, calculations for a Pt target at 300 V confirm that over 98% of the ejected species are individual Pt atoms (**Figure S2**). Additionally, the average kinetic energy of the ejected Pt atoms was found to be less than 10 eV which was calculated using force-field molecular dynamics. The average energy was derived from simulations of 480 collision events involving Ar atoms impacting random sites within an irreducible area of the Pt surface. Furthermore, threshold displacement energy of a carbon atom in graphene under the impact of a Pt atom was determined by *ab initio* molecular dynamic to be 38 eV, substantially higher than the observed Pt landing energies (<10 eV). Consequently, knock-on damage to the carbon lattice is not expected under our experimental conditions. Based on this information and our previous experimental results, we conclude that cluster formation occurs exclusively on the surface of the support material.

Building on this, we demonstrated that four key parameters dictate the stabilisation of single-layer metal clusters (SLMC) or bulk metal clusters (BMC) on support materials: metal coverage, flux of metal atoms, temperature, type, and concentration of defects. Among these factors, the generation of defects is the most significant, as they can strongly bind to metal atoms, thereby creating highly stable systems. Furthermore, if the concentration of defects can be well controlled, they could serve as selective traps for single metal atoms landing on surfaces, thus guiding their path on material surfaces. This approach could enable the formation of SLMC and BMS with precisely controlled sizes. However, defect generation also presents a significant challenge, as these defects are highly reactive and readily capture surrounding species, including oxygen molecules and moisture. In this work, we address this challenge by coupling argon ions irradiation with metal deposition onto support materials within the same chamber (**Figure S4**). This ensures that highly active defects are immediately available to bind with metal atoms. The experimental methodology used to achieve this is detailed in the next subsection, as well as the experimental results.


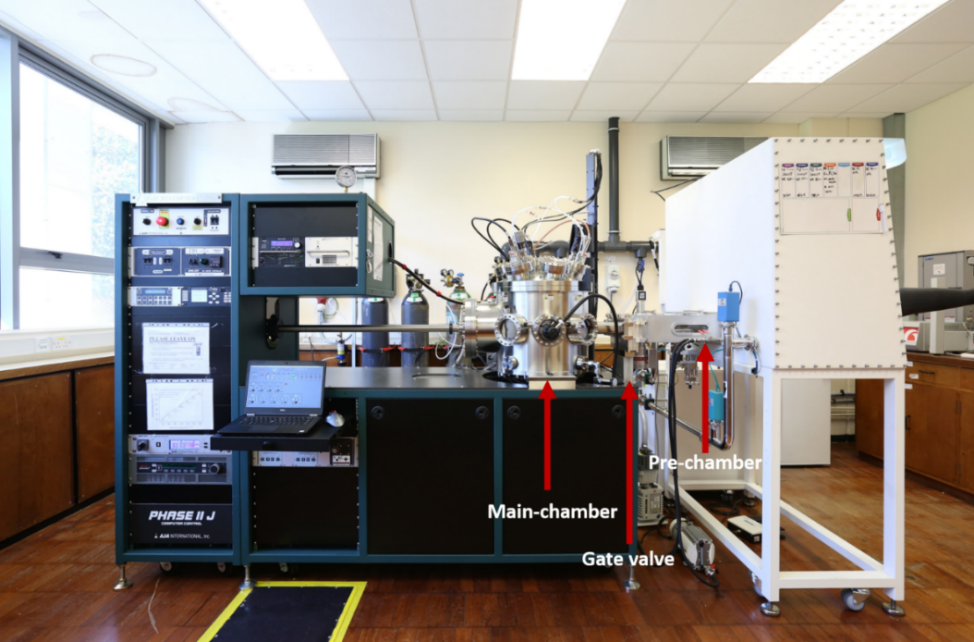


**Figure S4** | Photograph of the custom-designed AJA International magnetron sputtering system used in this study for powder surface treatment and metal atom deposition onto surface-treated powder materials.

In a typical experiment, the support materials (Vulcan XC 72R (CP), h-BN, GNF and GF) were placed in a custom-designed sample holder and loaded into the magnetron sputtering pre-chamber, where the background pressure reached 3 × 10⁻⁷ Torr within 15 minutes. The sample holder was then transferred to the main chamber, which had a background pressure of 3×10⁻⁸ Torr. After closing the gate valve, thereby isolating the main chamber from the pre-chamber, it took 5 minutes for the background pressure in the main chamber to stabilize at 3 × 10⁻⁸ Torr.

***4.2 Support characterisation after Argon ions irradiation***

Carbon surface treatment was performed using argon ions irradiation, with varying irradiation time and power levels as specified in Table S4. The changes in the carbon surface following argon ions irradiation were investigated using XPS. Below, we describe in more detail the C KLL, C 1s from XPS and the Raman analysis of the materials.

**Table S4:** Experimental parameters for argon surface treatment on Vulcan XC 72R support.

| **Support** | **Argon ions irradiation parameters** | | |
| --- | --- | --- | --- |
|  | Pressure | Power | time |
| Vulcan XC 72R | - | - | - |
| Vulcan XC 72R | 20 mTorr | 30W | 5s |
| Vulcan XC 72R | 20 mTorr | 30W | 15s |
| Vulcan XC 72R | 20 mTorr | 30W | 30s |
| Vulcan XC 72R | 20 mTorr | 30W | 60s |
| Vulcan XC 72R | 20 mTorr | 90W | 60s |

To obtain quantitative information about the concentration of defects on the Vulcan XC 72R surface after argon ion irradiation, we analysed the carbon Auger (C KLL) region. It has been demonstrated that by taking the first derivative of the carbon Auger spectrum and measuring the distance between the highest and lowest points, the D-parameter can be determined. This parameter exhibits a linear relationship with the *sp²*/*sp³* ratio at the material's surface, with the sp³ contribution correlating with an increased defect density (**Figure S5**). For the linear calibration of the D-parameters, we used reference values from the literature: 13.7 for pure sp³ carbon (diamond), as reported by A. Mezzi et al.^[1]^, and 23.5 for pure *sp²* carbon (HOPG cluster-cleaned), as reported by D. Morgan.^[2]^ The pristine sample, unexposed to argon irradiation, was labelled as CP, while the modified samples were labelled as CX%, where X corresponds to the sp³ content determined by D-parameter analysis (see Table S5).

**Table S5:** The D-parameter, sp³ percentage, and oxygen atomic content were derived from the first derivative of the C KLL Auger spectrum and the survey spectra of Vulcan XC 72R subjected to various argon ion irradiation treatments.

| Sample label | CP | C6% | C16% | C24% | C30% | C35% |
| --- | --- | --- | --- | --- | --- | --- |
| D-parameter | 23.1 | 22.9 | 21.9 | 21.1 | 20.5 | 20.1 |
| *sp^3^* % | 3.9 | 6.1 | 16.3 | 24.5 | 30.6 | 34.7 |
| O at% | 0.7 | 3.0 | 4.8 | 6.0 | 7.4 | 8.4 |


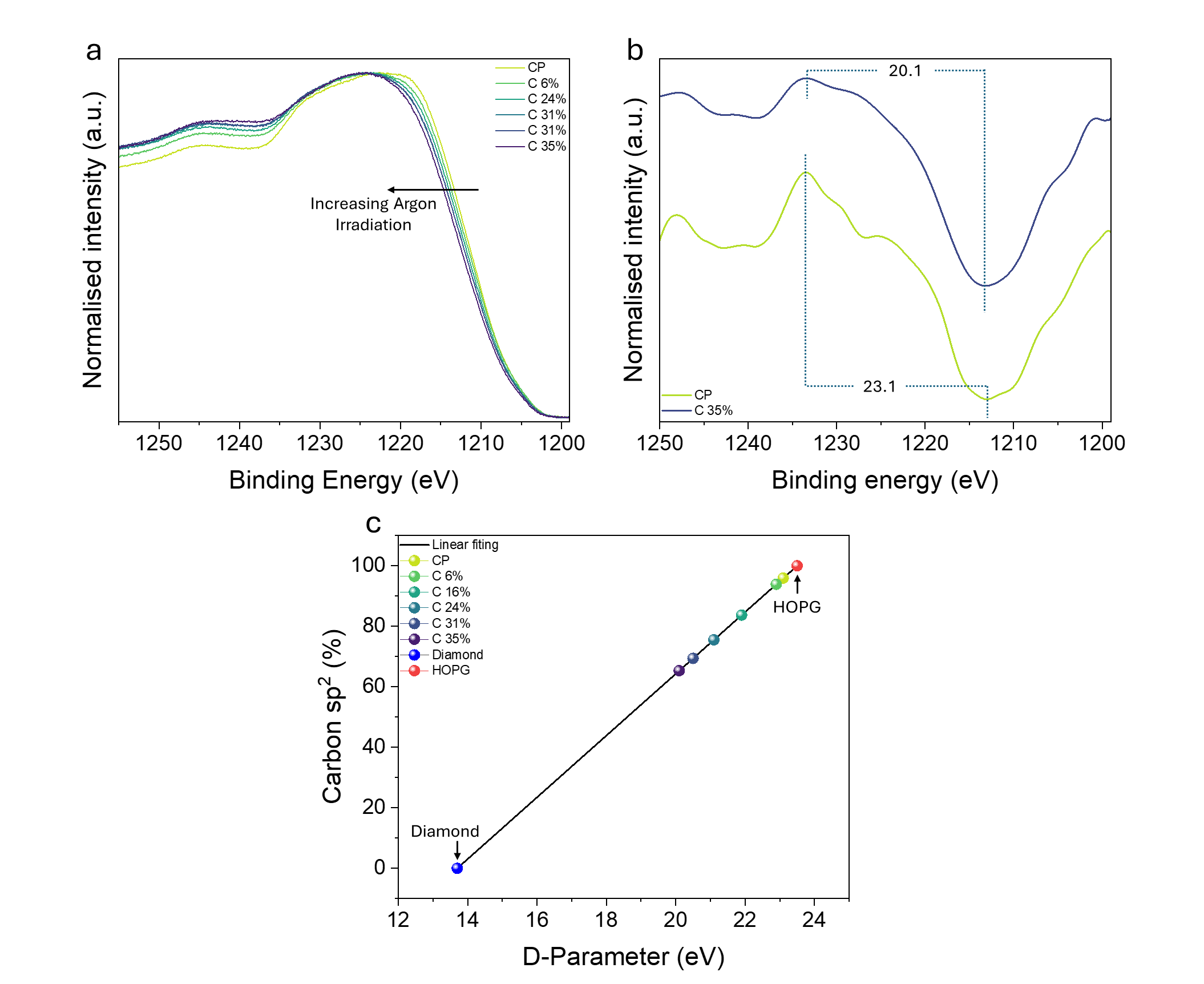


**Figure S5** | **C KLL spectra and D-parameter analysis for argon-irradiated carbon surfaces**. **a,** Normalized C KLL spectra for Vulcan XC 72R samples subjected to different argon ion irradiation. A gradual shift and broadening in spectral features are observed with increasing *sp^3^* content. **b,** First derivative of the C KLL for pristine carbon (CP) and the most irradiated sample (C 35%), highlighting the D-parameter, defined as the energy difference between the most prominent peaks (23.1 eV for CP and 20.1 eV for C 35%). **c,** Correlation between the D-parameter and the estimated surface sp³-carbon content (%), demonstrating a linear relationship. Reference materials (diamond and HOPG) are included for comparison. The D-parameter was extracted from the first derivative of the C KLL Auger spectrum. Samples were labelled according to the *sp³* percentage on the carbon surface, and details of the surface treatments are provided in Table S4.


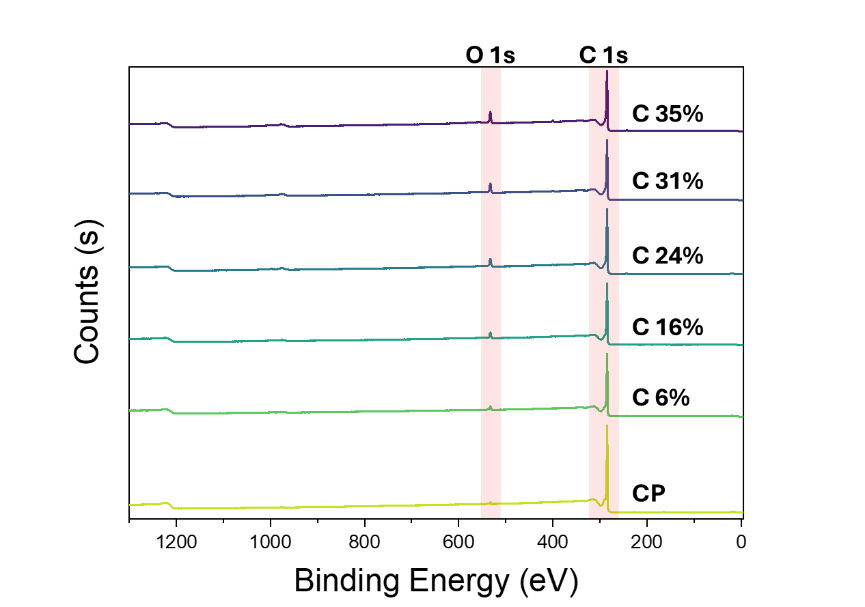


**Figure S6** | XPS survey spectra of Vulcan XC 72R (CP) with varying *sp^3^*% content. Highlight the C 1s region and the O 1s region, the later showing an increase in oxygen content as *sp^3^*% increases.

A symmetric peak shape characterises the graphitic carbon (*sp²* contribution). However, in the presence of different defects levels, the C 1s peak exhibits increased asymmetry towards the higher binding energy, with this effect becoming more pronounced as the surface treatment intensifies (**Figure S7a**). This shift can be correlated with an increase in the sp³ contribution, which is associated with a rise in the density of binding sites, such as carbon defects. Further insights into the structural changes in carbon were obtained by analysing the π-π* and shake-up satellite features in the C 1s spectra (**Figure S7b**). These loss features, typically observed in materials containing conjugated π-electron systems, are linked to the presence of aromatic rings within the graphitic network of carbon surface. A closer examination of the π-π* contribution in the C 1s region reveals a consistent decrease with increasing argon ion irradiation treatment time and power. Since the π-π* transitions are associated with the interactions between aromatic rings in the carbon structure, their diminishing intensity, and eventual disappearance, strongly suggests structural damage or increased disorder in the aromatic rings, likely due to carbon atom removal by argon ion irradiation of the carbon surface.


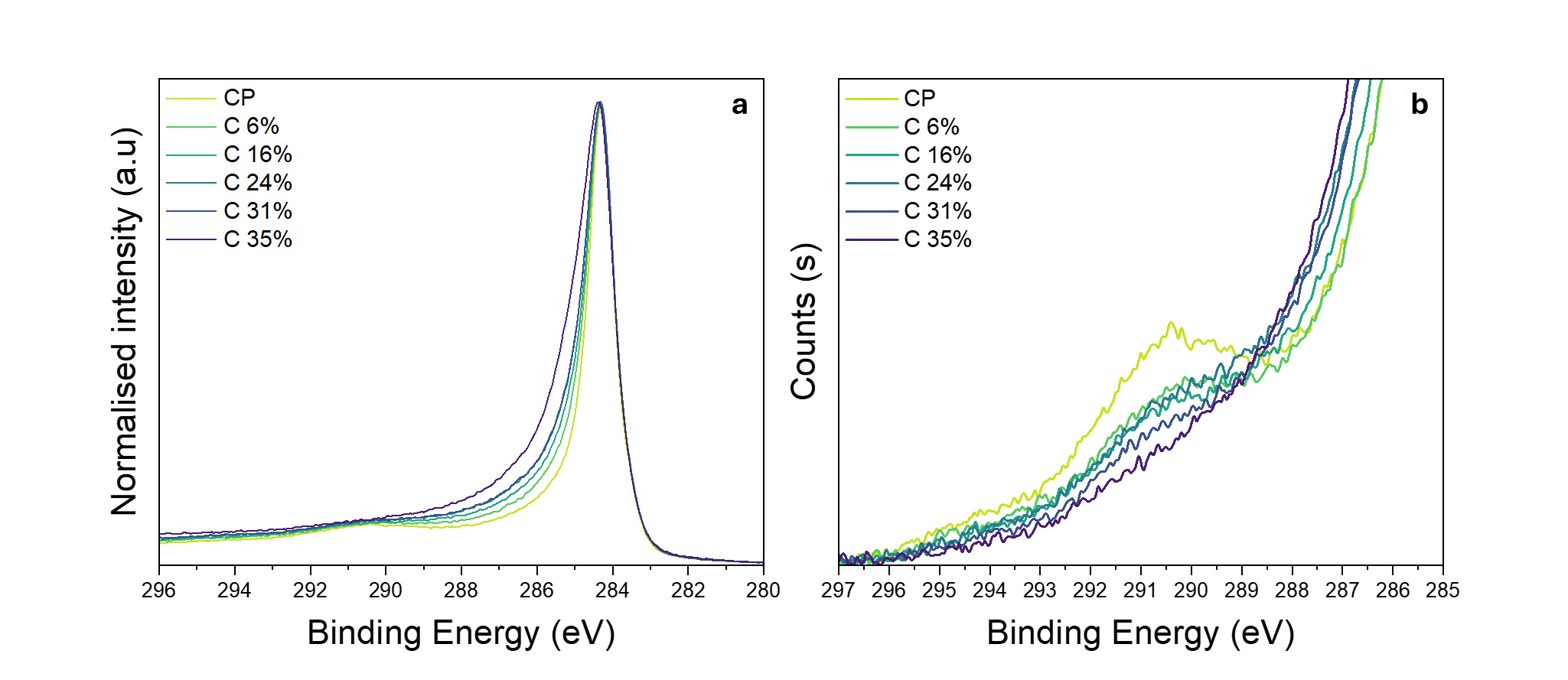


**Figure S7** | XPS spectra of Vulcan XC 72R irradiated with argon ions under different conditions. (a) High-resolution C 1s spectrum. (b) A zoomed-in view of the shake-up features in the C 1s region.

Argon ion irradiation of Vulcan XC 72R (at any time or power) decreases ID:IG relative to the reference sample, indicating these carbons - classified as stage two carbons (i.e., between nano-crystalline graphite and amorphous carbon) according to the "amorphisation trajectory" defined by Ferrari and Robertson - contain fewer aromatic rings, i.e., more structural defects. This would be broadly consistent with the observations from XPS, which suggest that argon ions irradiation results in an increase in the proportion of *sp^3^*-hybridised carbons, thus fewer aromatic rings. However, there is no obvious correlation between ID:IG and argon ions treatment time (as observed with XPS) and the differences in ID:IG (and thus La) between argon ions treated samples are very small. This may reflect (i) the reduced surface sensitivity of Raman spectroscopy relative to XPS and (ii) the reduced sampling volume (the lateral spot sizes in XPS and Raman spectroscopy are ~400 and ~1 μm, respectively) potentially yielding a skewed mean ID:IG if the sample is structurally heterogeneous and the sampling number is small.^[3]^


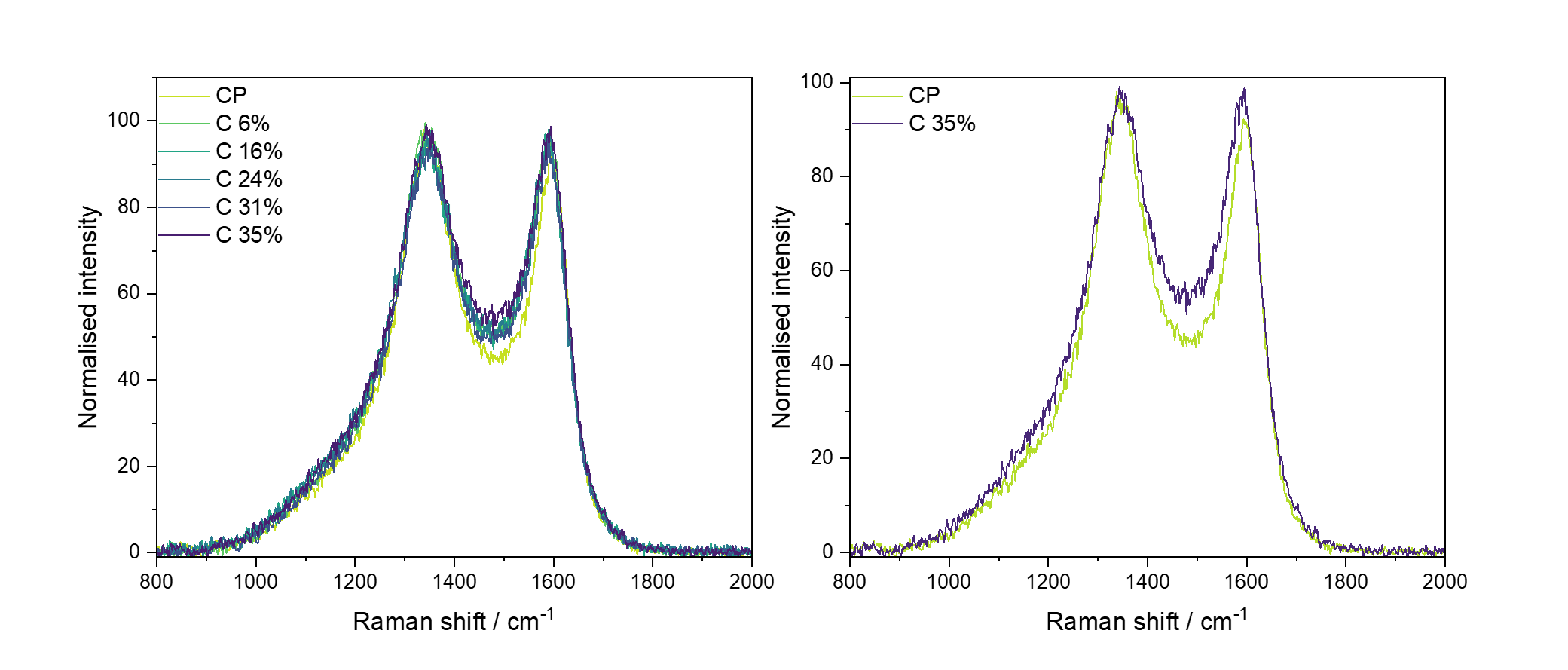


**Figure S8** | Representative 532 nm Raman spectra (left) of Vulcan XC 72R samples subjected to various argon ions irradiation conditions. The first order spectrum contains two strongly overlapping bands at ~1350 and 1590 cm-1, corresponding to the D (a ring breathing-mode of A1g symmetry) and G bands (an in-plane bond-stretching motion of pairs of sp2-hybridised carbon atoms of E2g symmetry), respectively. Additionally, the Raman spectra (right) of CP and C35% for direct comparation. All spectra have been baseline-corrected (using a linear model) and normalised to the intensity of the spectral maximum.

**Table S6:** Peak positions and relative intensities extracted from the 532 nm Raman spectra (N=3). I_D_:I_G_ is defined using peak heights, not areas. The cluster diameter, L_a_, is defined using I_D_:I_G_ = C’(λ)L_a_^2^, where C’(λ) is a wavelength-dependent coefficient.

| **Sample** | **D band / cm^-1^** | **G band / cm^-1^** | **I_D:_I_G_** | **L_a_ / nm** |
| --- | --- | --- | --- | --- |
| CP | 1347.5 ±0.5 | 1595.9±0.5 | 1.07 ±0.01 | 1.4 ±0.0 |
| C 6% | 1345.3 ±2.6 | 1591.2±4.4 | 1.03 ±0.01 | 1.4 ±0.0 |
| C 16% | 1345.6 ±1.4 | 1588.1±2.7 | 1.00 ±0.03 | 1.3 ±0.0 |
| C 24% | 1342.8 ±3.9 | 1586.7±2.6 | 1.01 ±0.02 | 1.4 ±0.0 |
| C 31% | 1348.4 ±1.0 | 1589.5±2.2 | 1.02 ±0.02 | 1.4 ±0.0 |
| C 35% | 1347.8 ±1.3 | 1591.2±1.7 | 0.99 ±0.01 | 1.3 ±0.0 |


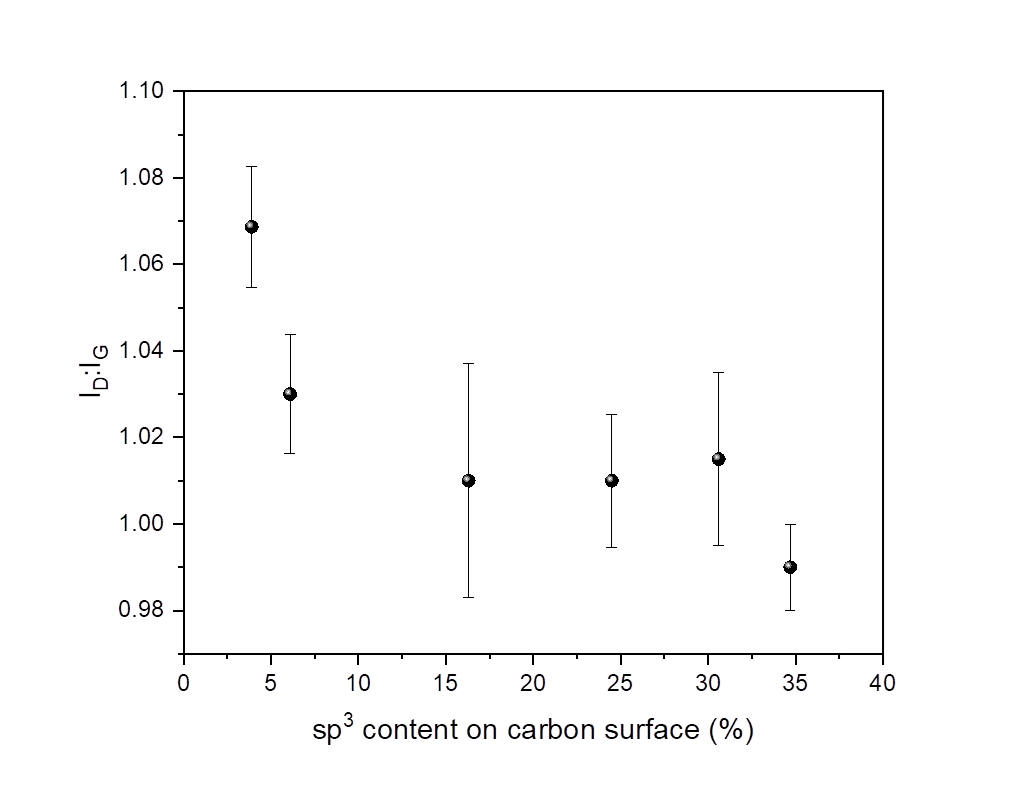


**Figure S9** | Correlation of I_D_:I_G_ between the samples with different *sp^3^* content.

***4.3 Metal atoms deposition onto Vulcan XC 72R with different binding sites densities***

After the surface treatment (**Table S4)**, metal atom deposition was conducted under a controlled working pressure, with various argon irradiation times and power settings applied. Once we found that the concentration of carbon *sp^3^* bonds increases with the argon ion irradiation conditions, we named the samples with the respective *sp^3^* percentage obtained from the C KLL XPS data. To understand how the *sp^3^* bond rise affects the nucleation and growth of metal atoms, we applied the same Pt deposition conditions on the previously studied samples. The experimental setup, analysis and images of these samples are shown in **Table S7 and S8** and **Supplementary Figures 10 and 11**.

**Table S7:** Experimental setup for the Surface optimisation of carbon support following the metal deposition for Vulcan XC 72R (C).

| Sample | Argon irradiation | | | Metal | Metal deposition | | | |
| --- | --- | --- | --- | --- | --- | --- | --- | --- |
|  | Pressure | Power | time |  | Pressure | Power | time | Working distance |
| Pt-CP | na | na | na | **Pt** | 10 mTorr | 15 W | **2 s** | 90 mm |
| Pt-C 6% | 20 mTorr | 30W | 5s | **Pt** | 10 mTorr | 15 W | **2 s** | 90 mm |
| Pt-C 16% | 20 mTorr | 30W | 15s | **Pt** | 10 mTorr | 15 W | **2 s** | 90 mm |
| Pt-C 24% | 20 mTorr | 30W | 30s | **Pt** | 10 mTorr | 15 W | **2 s** | 90 mm |
| Pt-C 31% | 20 mTorr | 30W | 60s | **Pt** | 10 mTorr | 15 W | **2 s** | 90 mm |
| Pt-C 35% | 20 mTorr | 90W | 60s | **Pt** | 10 mTorr | 15 W | **2 s** | 90 mm |

**Table S8:**  Summary of quantitative analysis extracted by the images on Supplementary Figs. 10 and 15.

| Support | Pt-CP | Pt-C6% | Pt-C16% | Pt-C24% | Pt-C31% | Pt-C35% | Pt-C35%-O |
| --- | --- | --- | --- | --- | --- | --- | --- |
| Argon irradiation | No | Yes | Yes | Yes | Yes | Yes | Yes |
| Exposed to air before deposition | no | no | no | no | no | no | Yes |
| Single-Atom | 50 | 106 | 180 | 233 | 259 | 305 | 133 |
| Dimers | 4 | 19 | 45 | 45 | 50 | 56 | 23 |
| Trimers | 7 | 9 | 18 | 22 | 24 | 19 | 9 |
| 2D Clusters | 10 | 26 | 32 | 38 | 70 | 61 | 25 |
| Average atoms per 2D Cluster | 16 ± 11 | 9 ± 6 | 9 ± 6 | 8 ± 4 | 9 ± 6 | 8 ± 4 | 7 ± 4 |
| 3D Clusters | 22 | 22 | 24 | 14 | 5 | 1 | 26 |
| Average atoms per 3D Cluster | 47 ± 36 | 29 ± 12 | 21 ± 12 | 14 ± 9 | 19 ± 2 | 15 | 39 ± 29 |
| Cluster diameter (nm) | 0.8 ± 0.2 | 0.7 ± 0.2 | 0.6 ± 0.1 | 0.5 ± 0.1 | 0.5 ± 0.1 | 0.5 ± 0.1 | 0.7 ± 0.2 |
| SLMC areal density (atom.nm^-2^) | 0.8 ± 0.5 | 1.6 ± 0.2 | 2.6 ± 0.4 | 3.1 ± 0.2 | 3.5 ± 0.3 | 4.3 ± 0.1 | 1.6 ± 0.3 |
| % of SLMC related to total Pt loading | 18.8% | 39.2% | 51.0% | 78.3% | 91.2% | 98.1% | 28% |


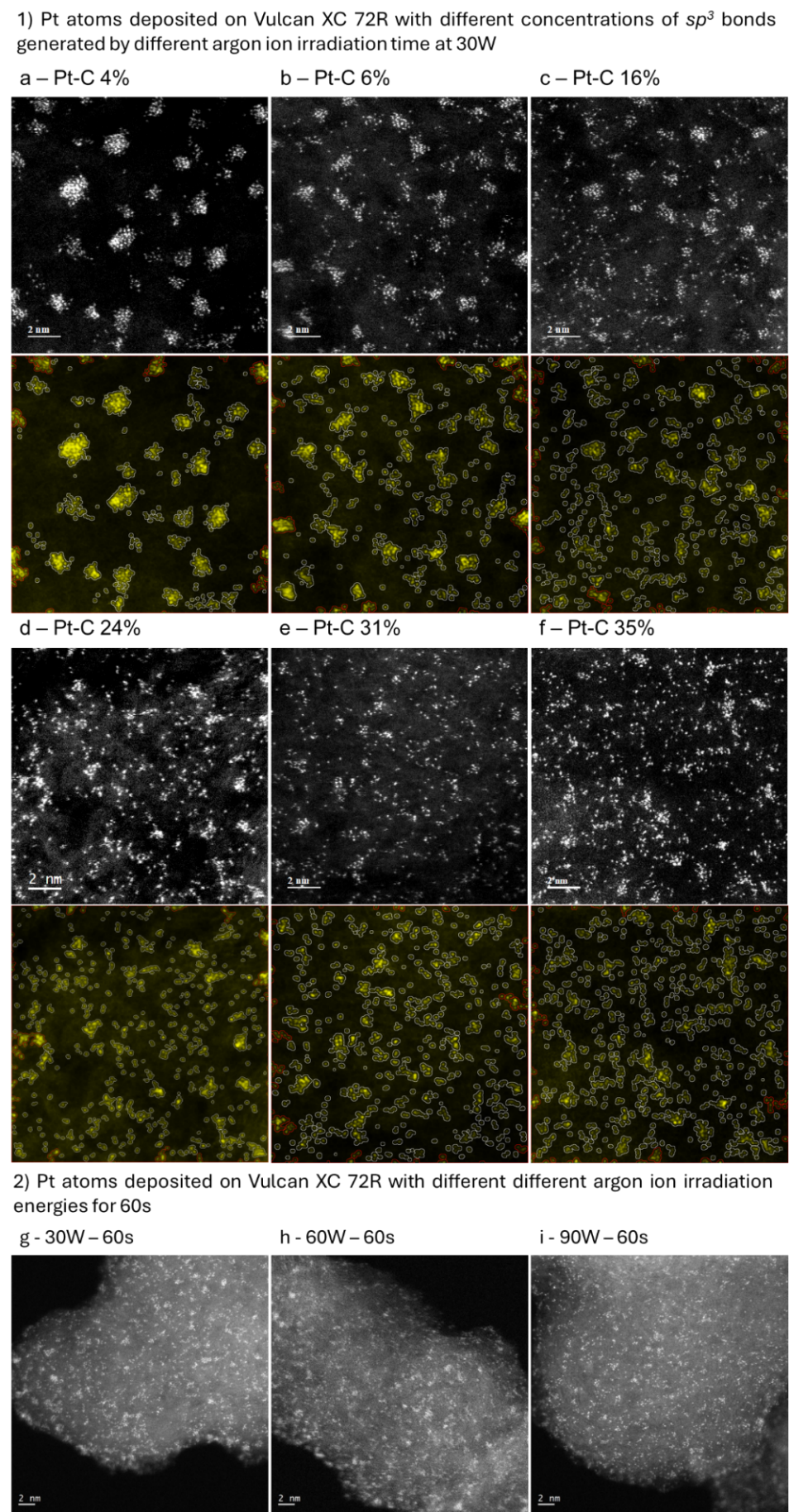


**Figure S10 |** AC-STEM and corresponding EDX elemental maps showing Pt atoms deposited on Vulcan XC-72R with varying concentrations of sp² bonds, generated by argon ion irradiation at 20 W for different durations. The C–Pt atomic dispersion increases progressively with the sp² content: (a) 4%, (b) 6%, (c) 16%, (d) 24%, (e) 31%, and (f) 35%. (2) AC-STEM images of Pt atoms deposited on Vulcan XC-72R subjected to different argon ion irradiation power settings (30 W, 60 W, and 90 W) for a fixed duration of 60 s, resulting in a SLMC % of 90.2, 94.9 and 99.1%, respectively.

**Table S9:** Literature comparison for Pt areal density on un-doped and doped carbon support.

| **Sample** | **Support information** | **Metal areal density (atoms/nm²)** | Ref. |
| --- | --- | --- | --- |
| Pt@C35% | Vulcan XC 72R | 4.3^1^ | This Work |
| Pt@C31% | Vulcan XC 72R | 3.5^1^ | This Work |
| Pt@C24% | Vulcan XC 72R | 3.1^1^ | This Work |
| Pt@C16% | Vulcan XC 72R | 2.6^1^ | This Work |
| Pt@C6% | Vulcan XC 72R | 1.6^1^ | This Work |
| Pt@C | Vulcan XC 72R | 0.8^1^ | This Work |
| Pt/CB | Carbon black BP2000 | 0.4^2^ | J. Am. Chem. Soc. 2023, 145, 13788−13795 |
| Pt/GQD | Graphene Quantum Dots | 0.5^2^ | J. Am. Chem. Soc. 2023, 145, 13788−13795 |
| Pt₁/BP_defect_ | Carbon black BP2000 | 0.02^2^ | Angewandte Chemie, 2018, 131, 4, 1175 |
| Pt1/p-NC@CNTs | porous N-doped carbons | 0.013^2^ | Adv. Funct. Mater. 2023, 33, 2307643 |
| Pt/NC | N-doped carbon | 0.08^2^ | Nat. Commun., 2019, 10, 1278 |
| Pt_N@rGO | N-doped reduced graphene oxide | 0.5^2^ | ACS Nano 2023, 17, 23, 23347–23358 |
| Pt/VC | Cl-doped Vulcan Carbon Black | 0.2 | ACS Appl. Nano Mater. 2024, 7, 18, 22011–22021 |
| Pt/NC | N-Doped carbon | 0.4^1,2^ | Nat. Nanotech., 2022, 17, 174–181 |
| Pt-N/BP | N-Doped Carbon Black BP2000 | 0.01^2^ | Nature Communications, 2017, 8, 15938 |
| Pt-PANI | N-doped carbon | ~3^1^ | ACS Catal. 2023, 13, 14, 9558–9566 |
| Pt/meso S-C | S-doped carbons | 0.9^2^ | Science Advances, 2019, 5, 10 |
| Pt/NC | N-doped carbon | 0.1^2^ | ACS Appl. Nano Mater. 2024, 7, 17, 20474–20483 |

^1^image analysis

^2^ICP/BET

The areal density comparisons are based on two widely used methodologies: (1) bulk measurements, which involve Inductively Coupled Plasma Optical Emission Spectroscopy (ICP-OES) for determining metal loading and BET (Brunauer-Emmett-Teller) surface area analysis, and (2) direct imaging via AC-STEM. Notably, Xiao Hai et al. (Nat. Nanotechnol., 2022, 17, 174–181) demonstrated that both methods yield highly consistent results for areal density, thereby validating either approach. In this work, we chose to use AC-STEM because it ensures the exclusion of 3D clusters, an essential requirement for a meaningful comparison between single-layer metal clusters (SLMC) and 3D clusters, and has been shown to produce results consistent with bulk methods.


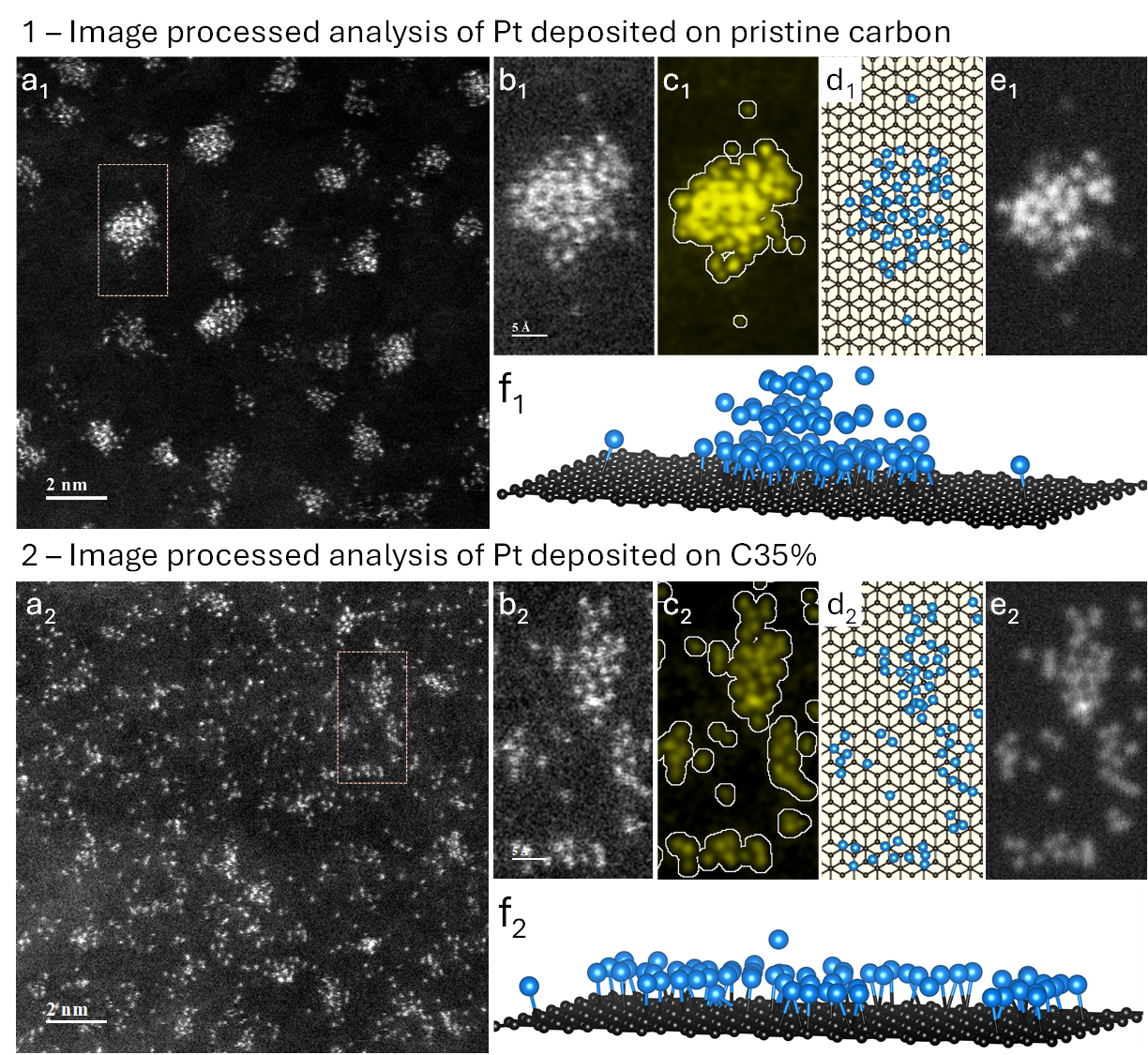


**Figure S11 | AC-STEM image analysis of Pt metal deposition on carbon supports.** (1) Pt deposited on pristine carbon (PC). (2) Pt deposited on C35%. Panels shows: a. Large-area raw AC-STEM images. b. Cropped regions highlighting individual Pt 3D clusters (1) and SLMC system (2). c. Image-processed regions from (b) with enhanced contrast and edge detection for atomic structure identification. d. Top-view of the atomic models generated from the processed images in (c), overlaid on a graphitic carbon surface. e. Simulated AC-STEM images based on the atomic models in (d). f. Side-view representations of the atomic models, showing Pt (blue) on the top layer carbon substrate (black)

***4.4*** *Metal atoms deposition onto graphitised nanofibers, graphite flake, and hexagonal boron nitride supports after argon ion irradiation*

**Table S10:** Experimental setup for the Surface optimisation of carbon support following the metal deposition for Graphitic nanofibers (GNF), Graphite flakes (GF) and hexagonal boron nitride (h-BN).

|  | Argon ion irradiation | | | Metal | Metal deposition | | | |
| --- | --- | --- | --- | --- | --- | --- | --- | --- |
| Sample name | Pressure | Power | time |  | Pressure | Power | time | Working distance |
| Pt@GNF |  |  |  |  | 10 mTorr | 15 W | 2 s | 90 mm |
| Pt@GNF 90W/60s | 20 mTorr | 90W | 60s | Pt | 10 mTorr | 15 W | 2 s | 90 mm |
| Pt@GF |  |  |  |  | 10 mTorr | 15 W | 2 s | 90 mm |
| Pt@GF 90W/60s | 20 mTorr | 90W | 60s | Pt | 10 mTorr | 15 W | 2 s | 90 mm |
| Pt@h-BN |  |  |  |  | 10 mTorr | 15 W | 2 s | 90 mm |
| Pt@h-BN 90W/60s | 20 mTorr | 90W | 60s | Pt | 10 mTorr | 15 W | 2 s | 90 mm |


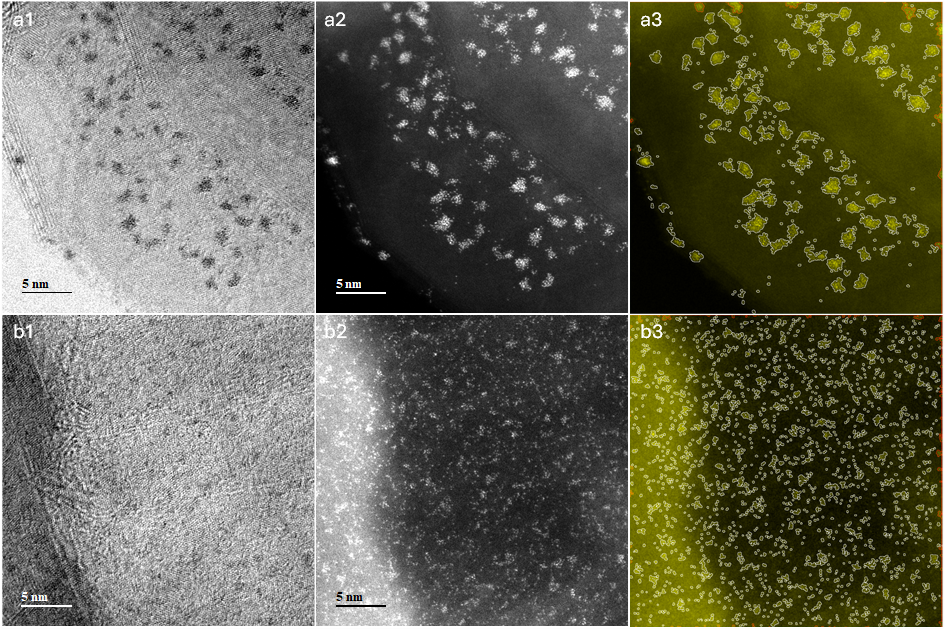


**Figure S12** | HRTEM (1), AC-STEM (2) and software image (3) tracking Pt SLMC and 3D cluster on pristine graphitise nanofibers (GNF) (a) and after argon irradiation (b).


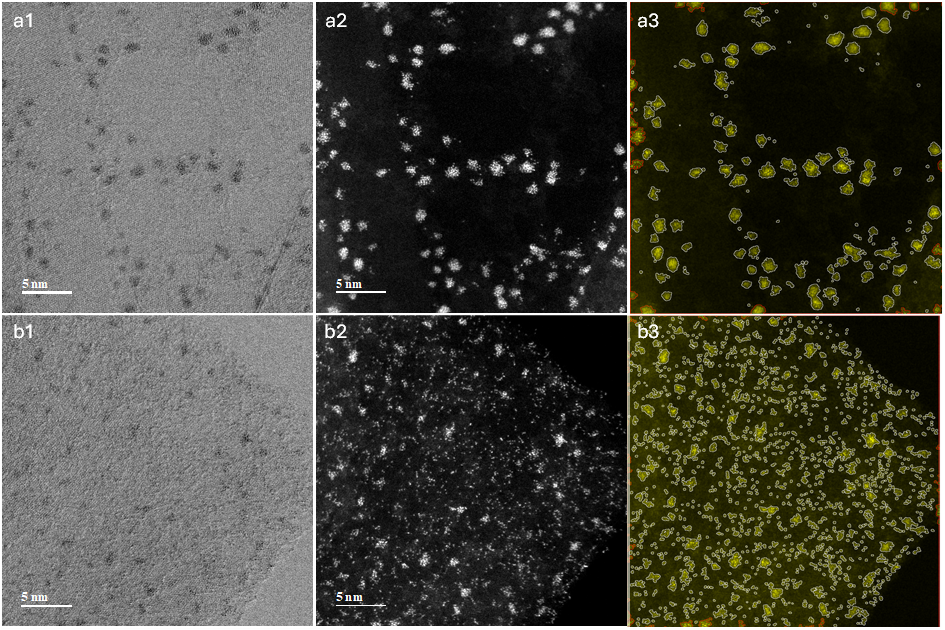


**Figure S13** | HRTEM (1), AC-STEM (2) and software image (3) tracking Pt SLMC and 3D cluster on pristine graphite flakes (GF) (a) and after argon irradiation (b).


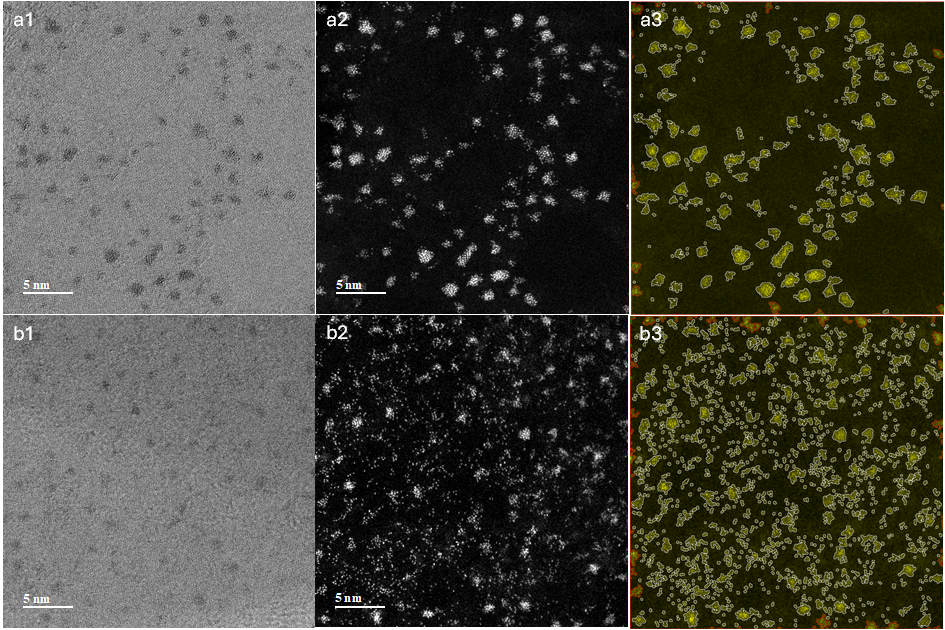


**Figure S14** | HRTEM (1), AC-STEM (2) and software image (3) tracking Pt SLMC and 3D cluster on pristine boron nitride (h-BN) (a) and after argon irradiation (b).

**Table S11:** Quantitative analysis for different supports obtained from the STEM images from different support with and without surface optimization. Image dimension 30.7 nm x 30.7 nm.

| Support | Ar^+^ | Number of SLMC | 3D Clusters | % of Pt atoms as SLMC |
| --- | --- | --- | --- | --- |
| GNF | No | 84 | 43 | 33% |
| GNF | Yes | 821 | 6 | 93% |
| GF | No | 60 | 30 | 27% |
| GF | Yes | 636 | 9 | 96% |
| h-BN | No | 144 | 94 | 12% |
| h-BN | Yes | 798 | 21 | 94% |

*4.5 Role of defect oxidation on the formation of SLMC*

**
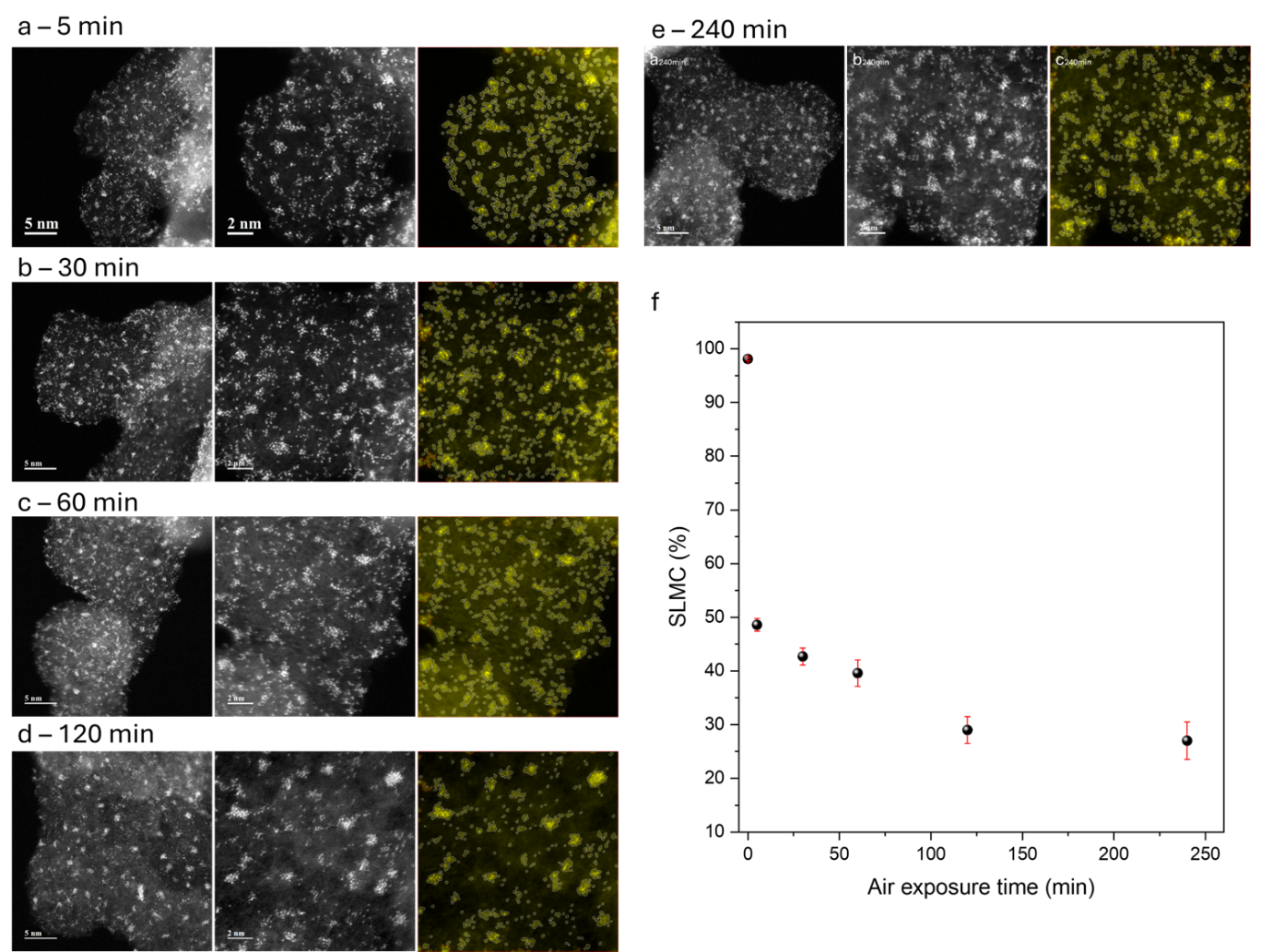
**

**Figure S15** | AC-STEM images and software images tracking Pt SLMC and 3D cluster on C35% that was exposed to air for 5 to 240 min (a-e) prior to Pt deposition. (f) shows the plot of the SLMC percentage against time exposure.

*4.6 Single-layer metal cluster library*

**Table S12:** Experimental setup for the Surface optimisation of carbon support following the deposition of different metals on Vulcan XC 72R (C) for the metal single-atom library.

| Sample | Argon ion irradiation | | | Metal | Metal deposition | | | |
| --- | --- | --- | --- | --- | --- | --- | --- | --- |
|  | Pressure | Power | time |  | Pressure | Power | time | Working distance |
| Ti@C | na | na | na | **Ti** | 10 mTorr | 16 W | **5 s** | 90 mm |
| Ti@C 35% | 20 mTorr | 90W | 60s | **Ti** | 10 mTorr | 16 W | **5 s** | 90 mm |
| V@C | na | na | na | **V** | 10 mTorr | 40 W | **3 s** | 90 mm |
| V@C 35% | 20 mTorr | 90W | 60s | **V** | 10 mTorr | 40 W | **3 s** | 90 mm |
| Cr@C | na | na | na | **Cr** | 10 mTorr | 15 W | **4 s** | 90 mm |
| Cr@C 35% | 20 mTorr | 90W | 60s | **Cr** | 10 mTorr | 15 W | **4 s** | 90 mm |
| Mn@C | na | na | na | **Mn** | 10 mTorr | 15 W | **2 s** | 90 mm |
| Mn@C 35% | 20 mTorr | 90W | 60s | **Mn** | 10 mTorr | 15 W | **2 s** | 90 mm |
| Fe@C | na | na | na | **Fe** | 10 mTorr | 30 W | **2 s** | 90 mm |
| Fe@C 35% | 20 mTorr | 90W | 60s | **Fe** | 10 mTorr | 30 W | **2 s** | 90 mm |
| Co@C | na | na | na | **Co** | 10 mTorr | 20 W | **2 s** | 90 mm |
| Co@C 35% | 20 mTorr | 90W | 60s | **Co** | 10 mTorr | 20 W | **2 s** | 90 mm |
| Ni@C | na | na | na | **Ni** | 10 mTorr | 20 W | **2 s** | 90 mm |
| Ni@C 35% | 20 mTorr | 90W | 60s | **Ni** | 10 mTorr | 20 W | **2 s** | 90 mm |
| Cu@C | na | na | na | **Cu** | 10 mTorr | 11 W | **3 s** | 90 mm |
| Cu@C 35% | 20 mTorr | 90W | 60s | **Cu** | 10 mTorr | 11 W | **3 s** | 90 mm |
| Zr@C | na | na | na | **Zr** | 10 mTorr | 25 W | **2 s** | 90 mm |
| Zr@C 35% | 20 mTorr | 90W | 60s | **Zr** | 10 mTorr | 25 W | **2 s** | 90 mm |
| Nb@C | na | na | na | **Nb** | 10 mTorr | 20 W | **4 s** | 90 mm |
| Nb@C 35% | 20 mTorr | 90W | 60s | **Nb** | 10 mTorr | 20 W | **4 s** | 90 mm |
| Mo@C | na | na | na | **Mo** | 10 mTorr | 15 W | **3 s** | 90 mm |
| Mo@C 35% | 20 mTorr | 90W | 60s | **Mo** | 10 mTorr | 15 W | **3 s** | 90 mm |
| Ru@C | na | na | na | **Ru** | 10 mTorr | 17 W | **2 s** | 90 mm |
| Ru@C 35% | 20 mTorr | 90W | 60s | **Ru** | 10 mTorr | 17 W | **2 s** | 90 mm |
| Pd@C | na | na | na | **Pd** | 10 mTorr | 12 W | **2 s** | 90 mm |
| Pd@C 35% | 20 mTorr | 90W | 60s | **Pd** | 10 mTorr | 12 W | **2 s** | 90 mm |
| Ag@C | na | na | na | **Ag** | 40 mTorr | 9 W | **2 s** | 80 mm |
| Ag@C 35% | 20 mTorr | 90W | 60s | **Ag** | 40 mTorr | 9 W | **2 s** | 80 mm |
| Sn@C | na | na | na | **Sn** | 20 mTorr | 15 W | **2 s** | 90 mm |
| Sn@C 35% | 20 mTorr | 90W | 60s | **Sn** | 20 mTorr | 15 W | **2 s** | 90 mm |
| Hf@C | na | na | na | **Hf** | 10 mTorr | 20 W | **2.5 s** | 90 mm |
| Hf@C 35% | 20 mTorr | 90W | 60s | **Hf** | 10 mTorr | 20 W | **2.5 s** | 90 mm |
| Ta@C | na | na | na | **Ta** | 20 mTorr | 20 W | **2 s** | 80 mm |
| Ta@C 35% | 20 mTorr | 90W | 60s | **Ta** | 20 mTorr | 20 W | **2 s** | 80 mm |
| W@C | na | na | na | **W** | 10 mTorr | 21 W | **2 s** | 90 mm |
| W@C 35% | 20 mTorr | 90W | 60s | **W** | 10 mTorr | 21 W | **2 s** | 90 mm |
| Re@C | na | na | na | **Re** | 20 mTorr | 20 W | **2 s** | 80 mm |
| Re@C 35% | 20 mTorr | 90W | 60s | **Re** | 20 mTorr | 20 W | **2 s** | 80 mm |
| Au@C | na | na | na | **Au** | 40 mTorr | 10 W | **2 s** | 70 mm |
| Au@C 35% | 20 mTorr | 90W | 60s | **Au** | 40 mTorr | 10 W | **2 s** | 70 mm |


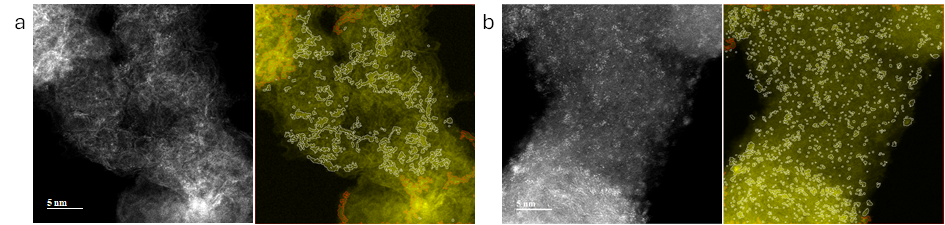


**Figure S16** | AC-STEM image and software image tracking of **Ti** SLMC and 3D cluster on Vulcan XC 72R without argon irradiation (a) and with argon irradiation (b).


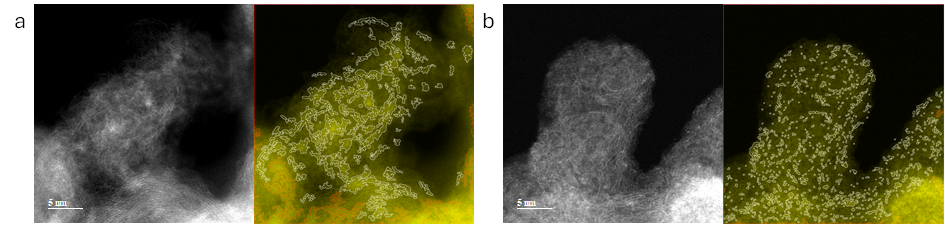


**Figure S17** | AC-STEM image and software image tracking of **V** SLMC and 3D cluster on Vulcan XC 72R without argon irradiation (a) and with argon irradiation (b).


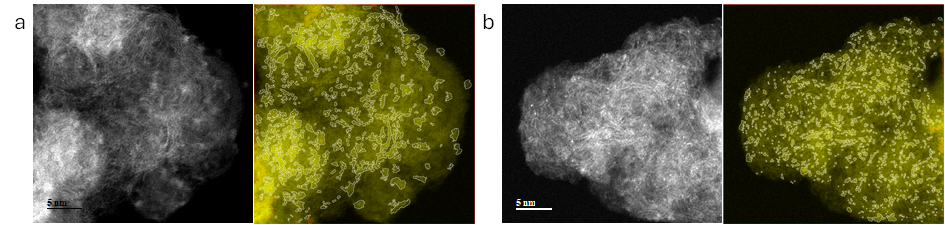


**Figure S18** | AC-STEM image and software image tracking of **Cr** SLMC and 3D cluster on Vulcan XC 72R without argon irradiation (a) and with argon irradiation (b).


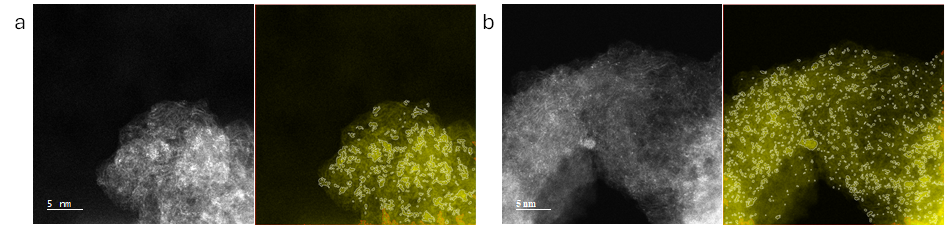


**Figure S19** | AC-STEM image and software image tracking of **Mn** SLMC and 3D cluster on Vulcan XC 72R without argon irradiation (a) and with argon irradiation (b).


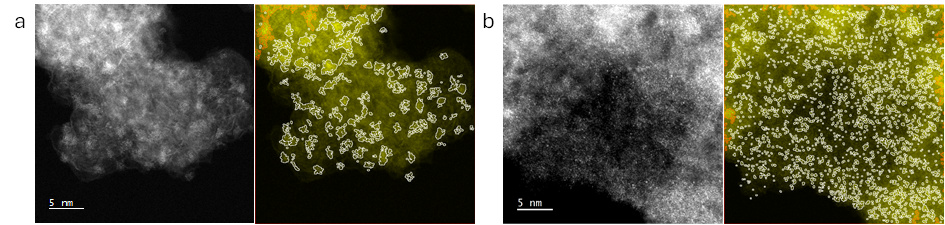


**Figure S20** | AC-STEM image and software image tracking of **Fe** SLMC and 3D cluster on Vulcan XC 72R without argon irradiation (a) and with argon irradiation (b).


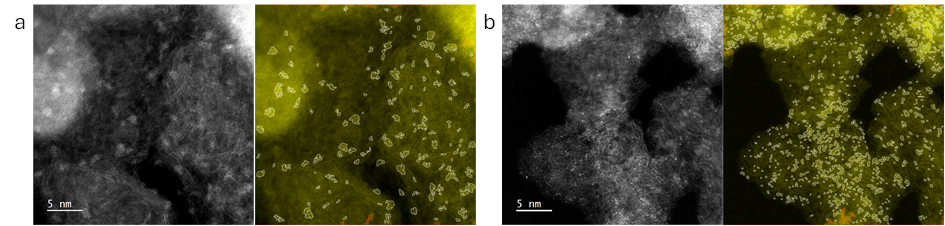


**Figure S21** | AC-STEM image and software image tracking of **Co** SLMC and 3D cluster on Vulcan XC 72R without argon irradiation (a) and with argon irradiation (b).


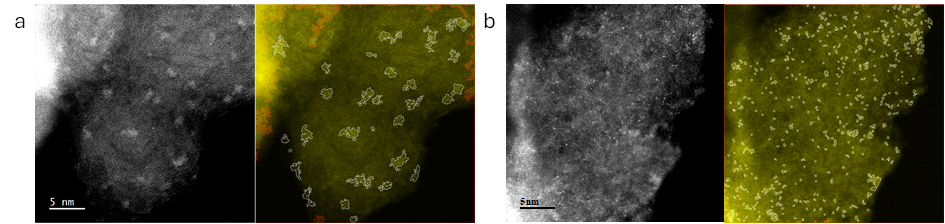


**Figure S22** | AC-STEM image and software image tracking of **Ni** SLMC and 3D cluster on Vulcan XC 72R without argon irradiation (a) and with argon irradiation (b).


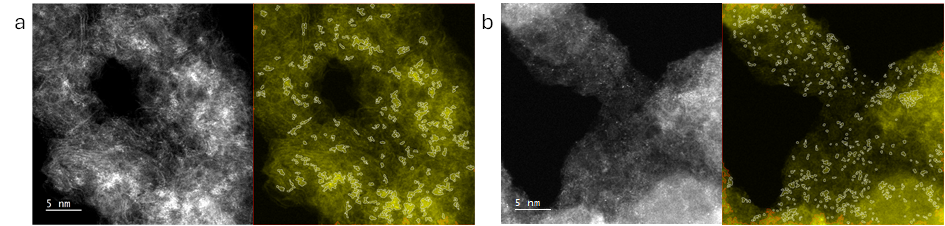


**Figure S23** | AC-STEM image and software image tracking of **Cu** SLMC and 3D cluster on Vulcan XC 72R without argon irradiation (a) and with argon irradiation (b).


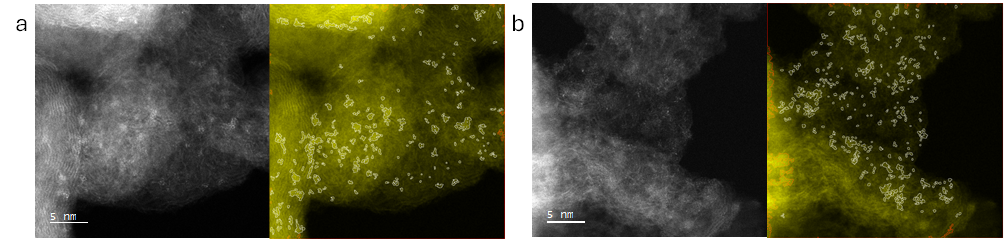


**Figure S24** | AC-STEM image and software image tracking of **Zr** SLMC and 3D cluster on Vulcan XC 72R without argon irradiation (a) and with argon irradiation (b).


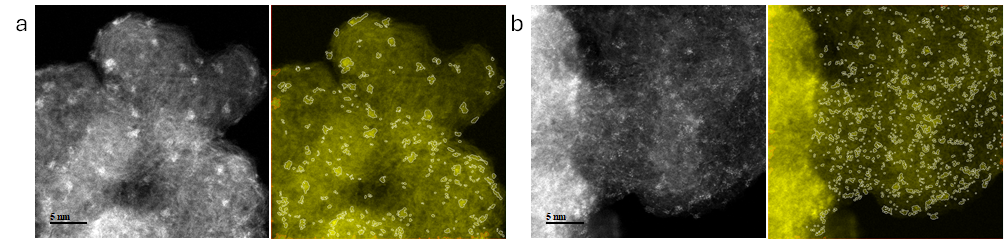


**Figure S25** | AC-STEM image and software image tracking of **Nb** SLMC and 3D cluster on Vulcan XC 72R without argon irradiation (a) and with argon irradiation (b).


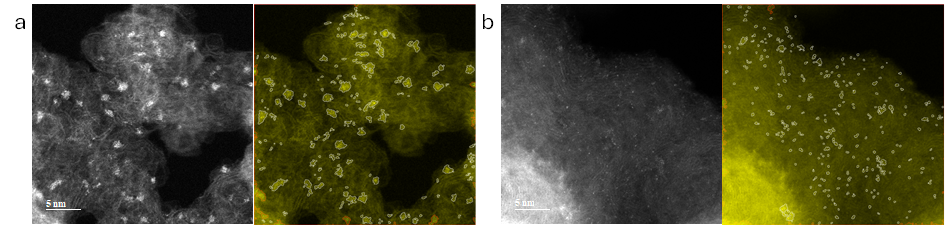


**Figure S26** | AC-STEM image and software image tracking of **Mo** SLMC and 3D cluster on Vulcan XC 72R without argon irradiation (a) and with argon irradiation (b).


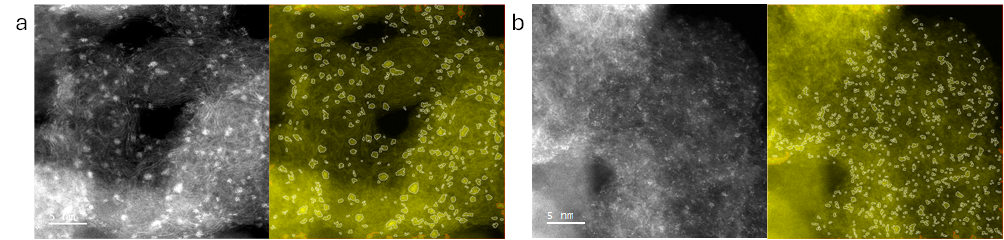


**Figure S27** | AC-STEM image and software image tracking of **Ru** SLMC and 3D cluster on Vulcan XC 72R without argon irradiation (a) and with argon irradiation (b).


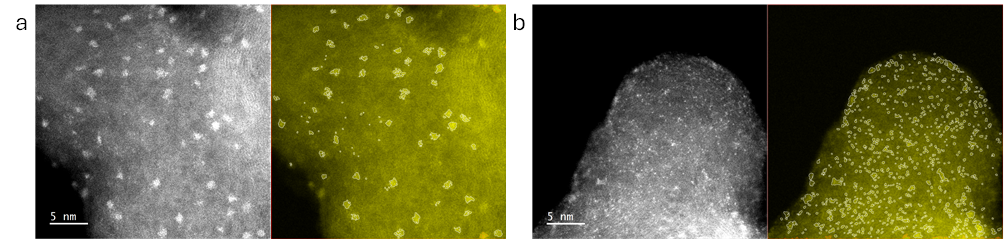


**Figure S28** | AC-STEM image and software image tracking of **Pd** SLMC and 3D cluster on Vulcan XC 72R without argon irradiation (a) and with argon irradiation (b).


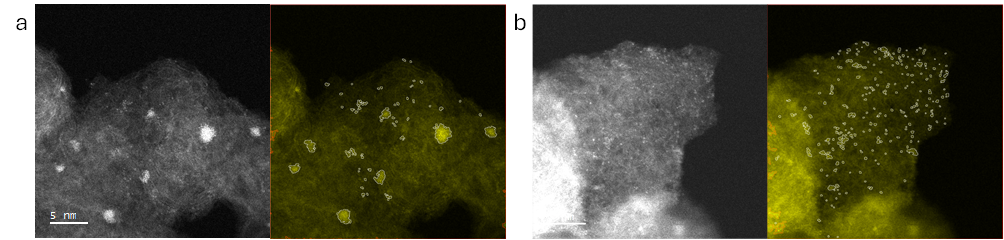


**Figure S29** |: AC-STEM image and software image tracking of **Ag** SLMC and 3D cluster on Vulcan XC 72R without argon irradiation (a) and with argon irradiation (b).


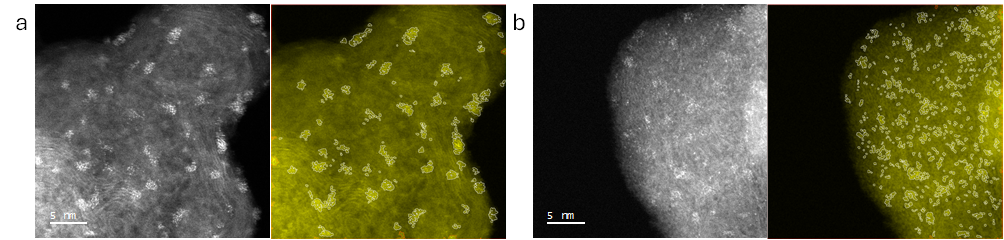


**Figure S30** | AC-STEM image and software image tracking of **Sn** SLMC and 3D cluster on Vulcan XC 72R without argon irradiation (a) and with argon irradiation (b).


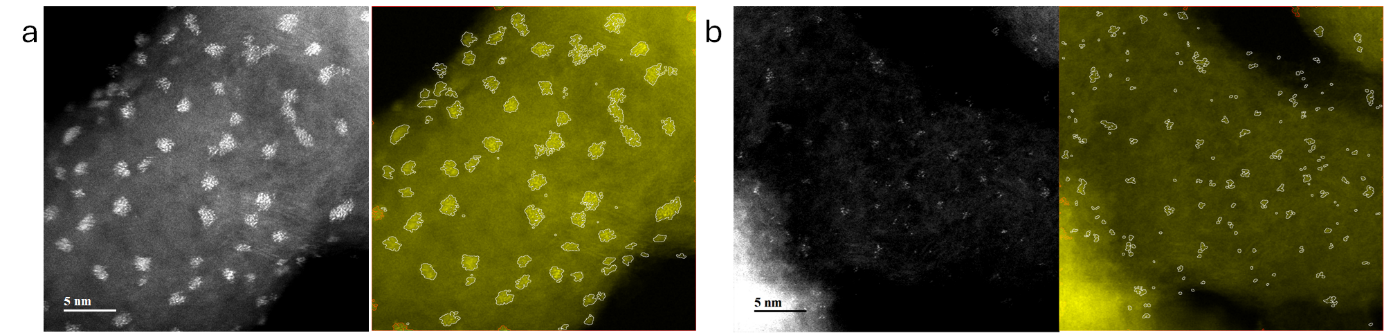


**Figure S31** | AC-STEM image and software image tracking of **Hf** SLMC and 3D cluster on Vulcan XC 72R without argon irradiation (a) and with argon irradiation (b).


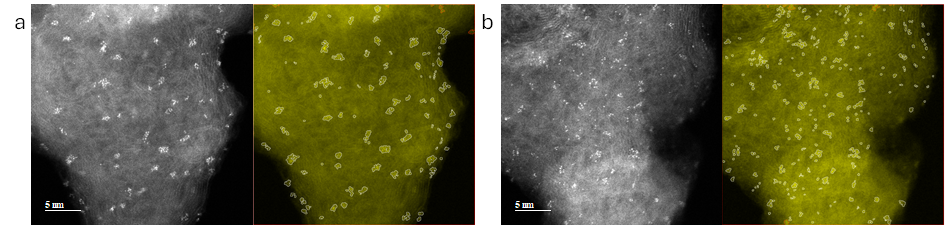


**Figure S32** | AC-STEM image and software image tracking of **Ta** SLMC and 3D cluster on Vulcan XC 72R without argon irradiation (a) and with argon irradiation (b).


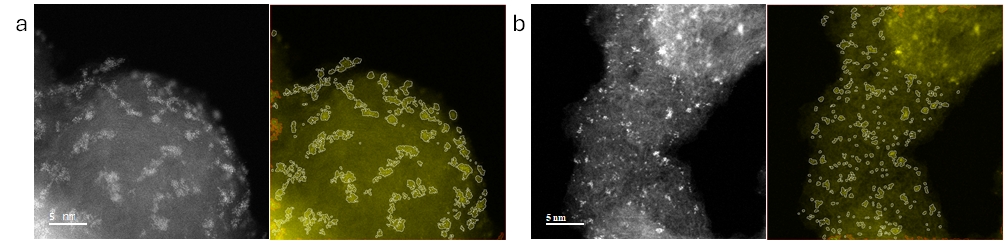


**Figure S33** | AC-STEM image and software image tracking of **W** SLMC and 3D cluster on Vulcan XC 72R without argon irradiation (a) and with argon irradiation (b).


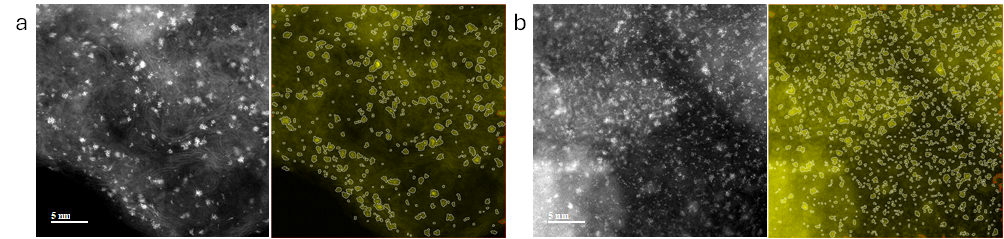


**Figure S34** | AC-STEM image and software image tracking of **Re** SLMC and 3D cluster on Vulcan XC 72R without argon irradiation (a) and with argon irradiation (b).


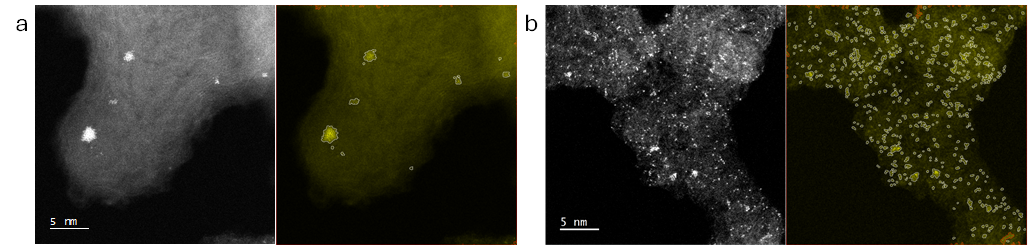


**Figure S35** | AC-STEM image and software image tracking of **Au** SLMC and 3D cluster on Vulcan XC 72R without argon irradiation (a) and with argon irradiation (b).

**Table S13:** Summary information obtained from quantitatively analysing STEM images using homemade Python code software.

| Metal | Ar^+^ | Number of SLMC | 3D Clusters | % of Pt atoms as SLMC | Areal Density (atoms/nm^2^) |
| --- | --- | --- | --- | --- | --- |
| Ti | No | 84 | 43 | 33% | 1.2 |
| Ti | Yes | 821 | 6 | 93% | 2.8 |
| V | No | 60 | 30 | 27% | 2.1 |
| V | Yes | 636 | 9 | 96% | 2.7 |
| Cr | No | 144 | 94 | 12% | 0.7 |
| Cr | Yes | 798 | 21 | 94% | 3.2 |
| Mn | No | 104 | 30 | 43% | 1.0 |
| Mn | Yes | 745 | 9 | 88% | 2.9 |
| Fe | No | 96 | 34 | 24% | 1.0 |
| Fe | Yes | 952 | 9 | 99% | 4.2 |
| Co | No | 65 | 72 | 16% | 0.3 |
| Co | Yes | 636 | 16 | 89% | 2.7 |
| Ni | No | 10 | 21 | 15% | 0.1 |
| Ni | Yes | 388 | 2 | 98% | 0.8 |
| Cu | No | 100 | 40 | 29% | 0.4 |
| Cu | Yes | 313 | 3 | 98% | 1.0 |
| Zr | No | 96 | 75 | 16% | 0.3 |
| Zr | Yes | 204 | 2 | 98% | 1.1 |
| Nb | No | 133 | 68 | 22% | 0.4 |
| Nb | Yes | 648 | 13 | 91% | 2.7 |
| Mo | No | 106 | 42 | 19% | 0.3 |
| Mo | Yes | 221 | 1 | 99% | 1.2 |
| Ru | No | 218 | 101 | 30% | 0.9 |
| Ru | Yes | 516 | 9 | 95% | 3.0 |
| Pd | No | 56 | 38 | 19% | 0.4 |
| Pd | Yes | 530 | 2 | 99% | 1.6 |
| Ag | No | 42 | 7 | 18% | 0.1 |
| Ag | Yes | 181 | 7 | 86% | 1.2 |
| Sn | No | 68 | 31 | 23% | 0.6 |
| Sn | Yes | 324 | 5 | 97% | 2.9 |
| Hf | No | 41 | 49 | 8% | 0.4 |
| Hf | Yes | 229 | 15 | 87% | 1.5 |
| Ta | No | 58 | 43 | 21% | 0.3 |
| Ta | Yes | 213 | 3 | 96% | 0.8 |
| W | No | 83 | 53 | 13% | 0.8 |
| W | Yes | 269 | 16 | 71% | 1.7 |
| Re | No | 271 | 115 | 25% | 0.6 |
| Re | yes | 591 | 30 | 82% | 3.3 |
| Au | No | 6 | 5 | 3% | 0.02 |
| Au | Yes | 361 | 5 | 90% | 1.5 |

**Table S14:** Summary of the oxidation state of the single metal atom library, highlighting the XPS signal and binding energy reference.

|  | Metal | XPS Signal | Binding energy (eV) | Oxidation state | Fitting model ref |
| --- | --- | --- | --- | --- | --- |
| 1^st^ Row | Ti | 2p 3/2 | 460.1 | +4 | ^[4]^ |
|  | V | 2p 3/2 | 517.3 | +5 | ^[4]^ |
|  | Cr | 2p 3/2 | 577.2 | +3 | ^[5]^ |
|  | Mn | 2p 3/2 | 641.2 | +2 | ^[5]^ |
|  | Fe | 2p 3/2 | 709.7 | +3 | ^[6]^ |
|  | Co | 2p 3/2 | 780.5 | +2 | ^[5]^ |
|  | Ni | 2p 3/2 | 854.9 | +2 | ^[7]^ |
|  | Cu | 2p 3/2 | 932.7 | +1 | ^[8]^ |
| 2^nd^ Row | Zr | 3d 5/2 | 182.8 | +4 | ^[9]^ |
|  | Nb | 3d 5/2 | 207.4 | +5 | ^[10]^ |
|  | Mo | 3d 5/2 | 232.6 | +6 | ^[11]^ |
|  | Ru | 3p 5/2 | 465.7 | +4 | ^[12]^ |
|  | Pd | 3d 5/2 | 337.0 | +2 | ^[13]^ |
|  | Ag | 3d 5/2 | 368.6 | +1 | ^[14]^ |
|  | Sn | 3d 5/2 | 486.8 | +4 | ^[15]^ |
| 3^rd^ Row | Hf | 4f 7/2 | 17.6 | +4 | ^[16]^ |
|  | Ta | 4f 7/2 | 26.5 | +5 | ^[17]^ |
|  | W | 4f 7/2 | 35.9 | +6 | ^[18]^ |
|  | Re | 4f 7/2 | 45.5 | +6 | ^[19]^ |
|  | Pt | 4f 7/2 | 72.6 | +2 | ^[20]^ |
|  | Au | 4f 7/2 | 84.5 | 0 | ^[21]^ |


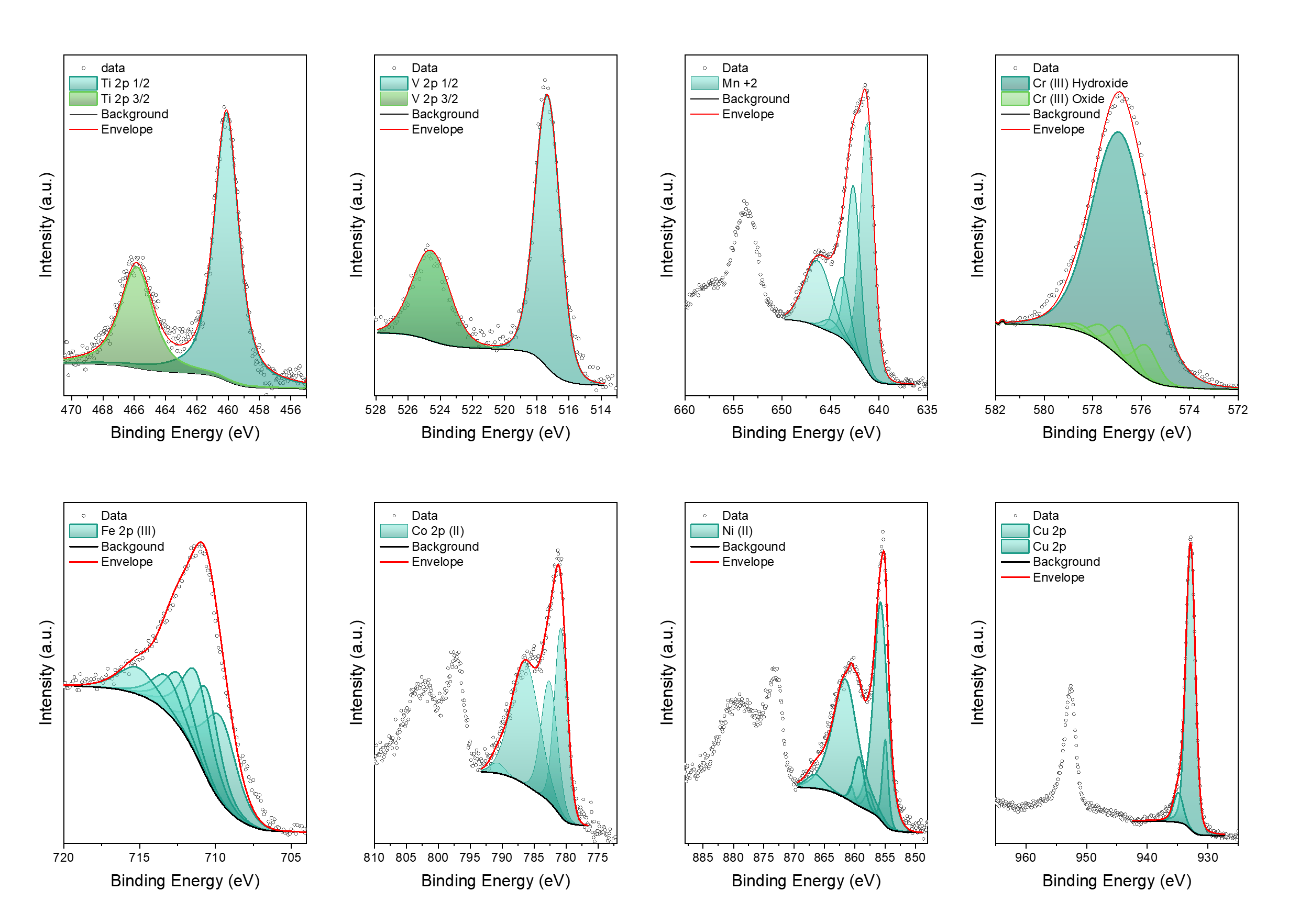


**Figure S36** | XPS of the first row of transition metals for the single-layer metal cluster library.


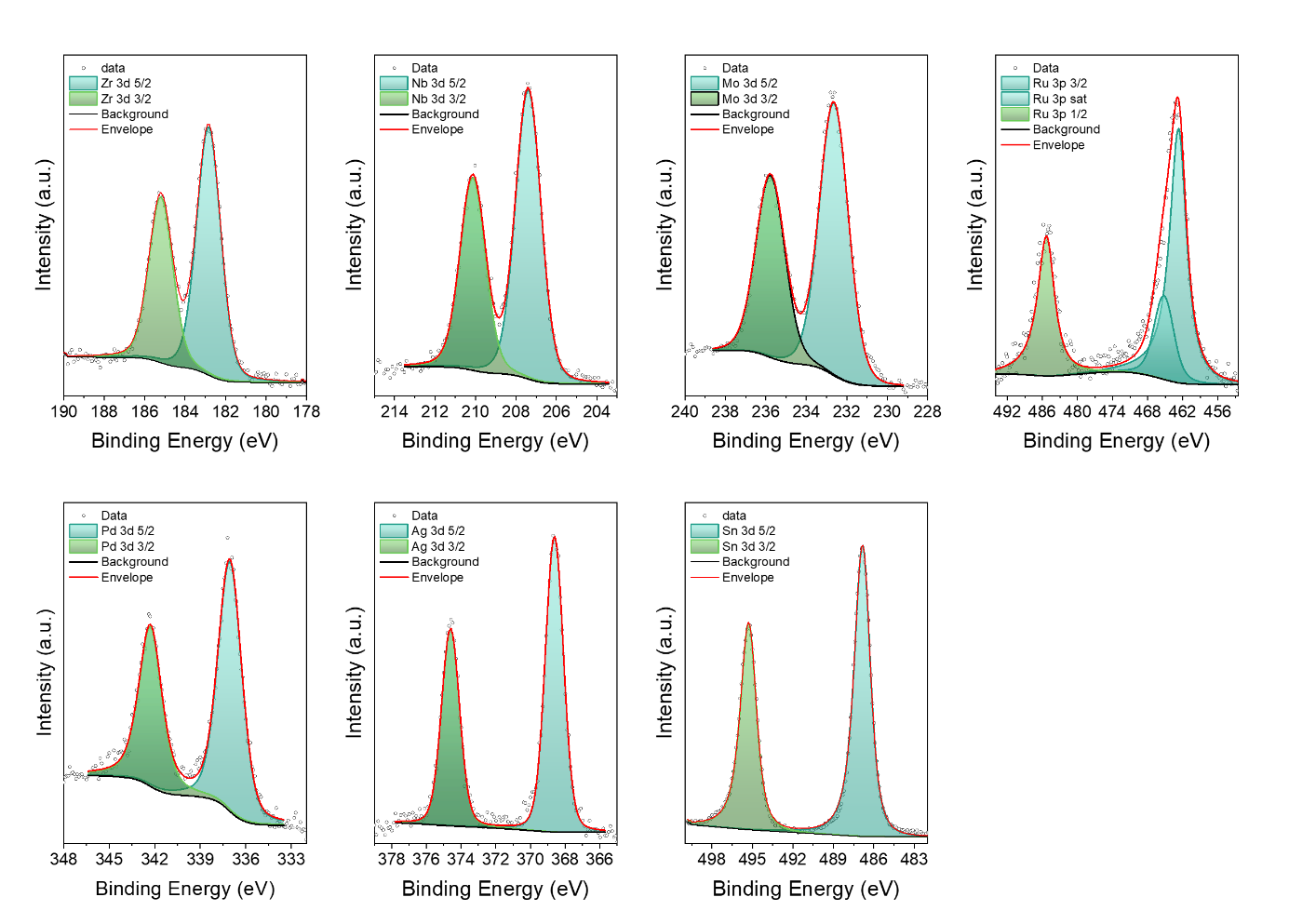


**Figure S37** | XPS of the second row of transition metals for the single-layer metal cluster library.


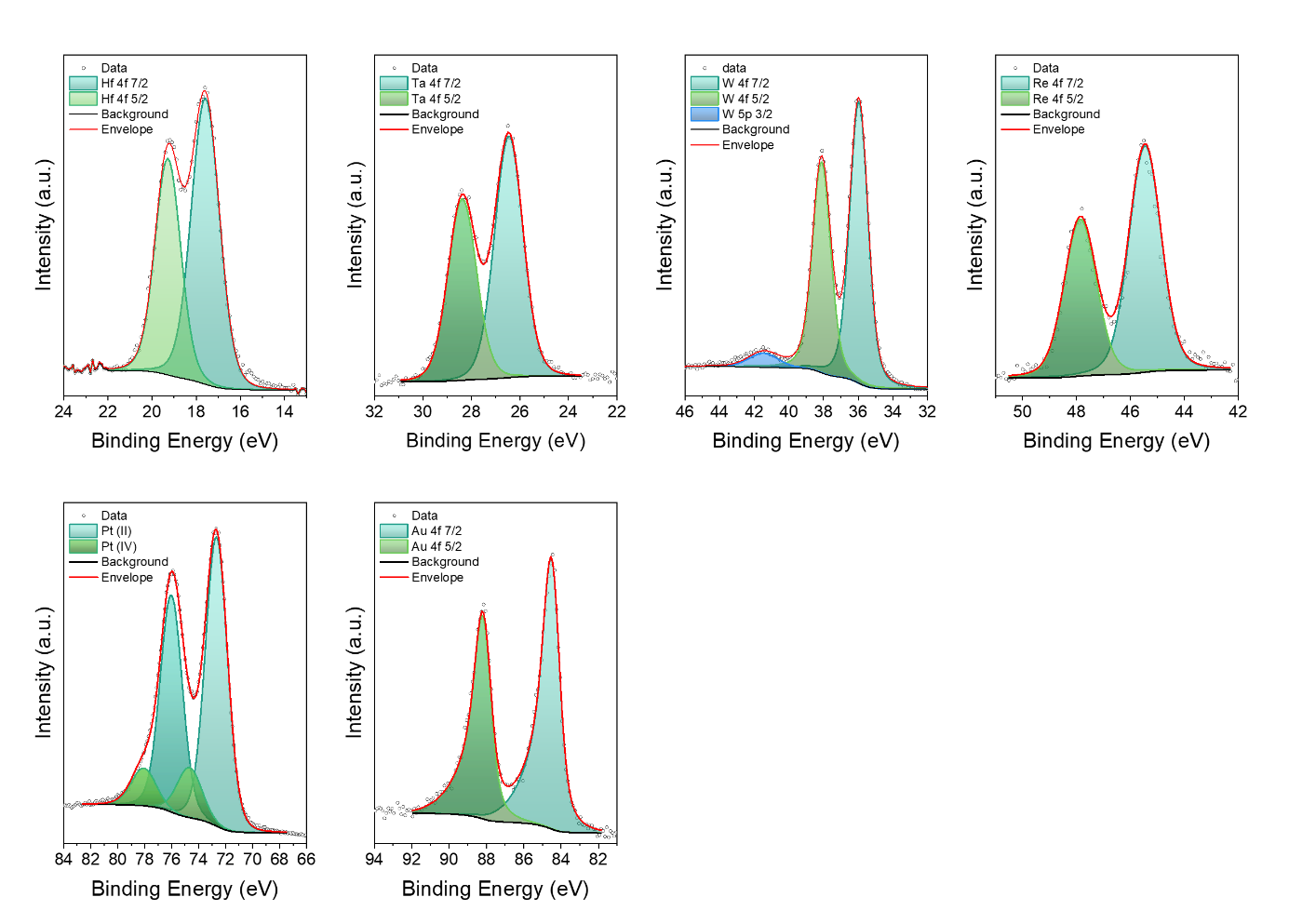


**Figure S38** | XPS of the third row of transition metals for the single-layer metal cluster library.

*4.7 Multimetallic single-layer metal alloy cluster*

**Table S15:** Experimental setup for the Surface optimisation of carbon support following the deposition of different metals on Vulcan XC 72R (C) for the multimetallic system**.**

| Sample | Argon ion irradiation | | | Metal | Metal deposition | | | |
| --- | --- | --- | --- | --- | --- | --- | --- | --- |
|  | Pressure | Power | time |  | Pressure | Power | time | Working distance |
| NiPd@C | na | na | na | **Ni** | 20 mTorr | 22 W | **2.5 s** | 80 mm |
|  |  |  |  | **Pd** | 20 mTorr | 12 W | **2.5 s** | 80 mm |
| NiPd@C 35% | 20 mTorr | 90W | 60s | **Ni** | 20 mTorr | 22 W | **2.5 s** | 80 mm |
|  |  |  |  | **Pd** | 20 mTorr | 12 W | **2.5 s** | 80 mm |
| NiPt@C | na | na | na | **Ni** | 20 mTorr | 22 W | **2.5 s** | 80 mm |
|  |  |  |  | **Pt** | 20 mTorr | 15 W | **2.5 s** | 80 mm |
| NiPt@C 35% | 20 mTorr | 90W | 60s | **Ni** | 20 mTorr | 22 W | **2.5 s** | 80 mm |
|  |  |  |  | **Pt** | 20 mTorr | 15 W | **2.5 s** | 80 mm |
| PdPt@C | na | na | na | **Pd** | 20 mTorr | 12 W | **2.5 s** | 80 mm |
|  |  |  |  | **Pt** | 20 mTorr | 15 W | **2.5 s** | 80 mm |
| PdPt@C 35% | 20 mTorr | 90W | 60s | **Pd** | 20 mTorr | 12 W | **2.5 s** | 80 mm |
|  |  |  |  | **Pt** | 20 mTorr | 15 W | **2.5 s** | 80 mm |
| NiPdPt@C | na | na | na | **Ni** | 40 mTorr | 22 W | **2.5 s** | 80 mm |
|  |  |  |  | **Pd** | 40 mTorr | 12 W | **2.5 s** | 80 mm |
|  |  |  |  | **Pt** | 40 mTorr | 15 W | **2.5 s** | 80 mm |
| NiPdPt@C 35% | 20 mTorr | 90W | 60s | **Ni** | 40 mTorr | 22 W | **2.5 s** | 80 mm |
|  |  |  |  | **Pd** | 40 mTorr | 12 W | **2.5 s** | 80 mm |
|  |  |  |  | **Pt** | 40 mTorr | 15 W | **2.5 s** | 80 mm |

**Figure S39** | **Summary of** **Multimetallic systems on carbon surfaces with and without surface engineering.** **a**, AC-STEM images of bi- and trimetallic alloys on pristine carbon surfaces, showing the formation of 3D clusters with large empty regions. **b**, AC-STEM images reveal that after argon ion irradiation, SLMC alloys predominantly form, with no large empty regions.


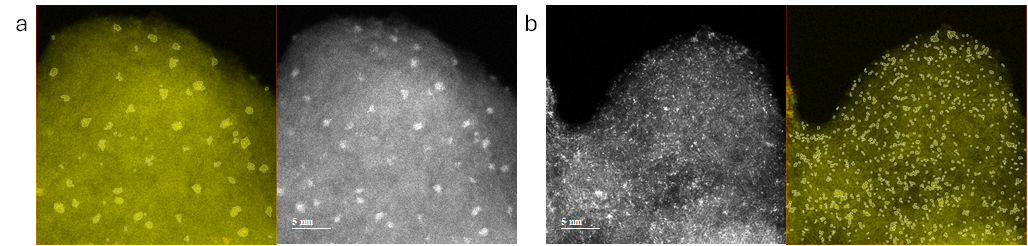


**Figure S40** | AC-STEM image and software image tracking of bimetallic **NiPd** SLMC and 3D cluster on Vulcan XC 72R without argon irradiation (a) and with argon irradiation (b).


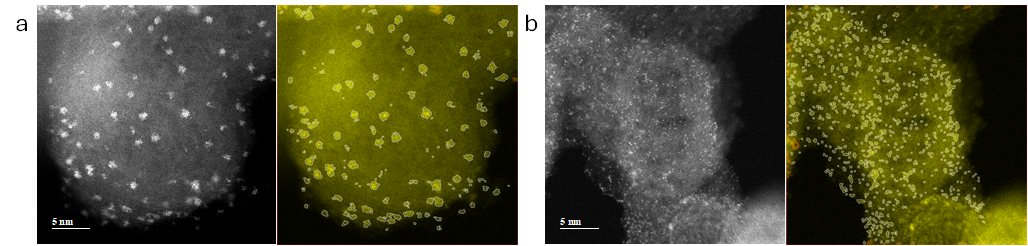


**Figure S41** | AC-STEM image and software image tracking of bimetallic **NiPt** SLMC and 3D cluster on Vulcan XC 72R without argon irradiation (a) and with argon irradiation (b).


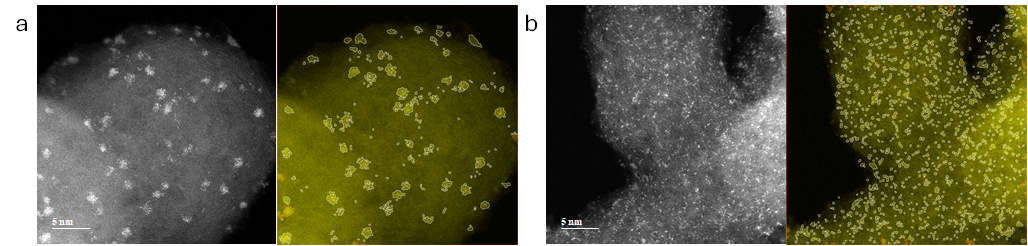


**Figure S42** | AC-STEM image and software image tracking of bimetallic **PdPt** SLMC and 3D cluster on Vulcan XC 72R without argon irradiation (a) and with argon irradiation (b).


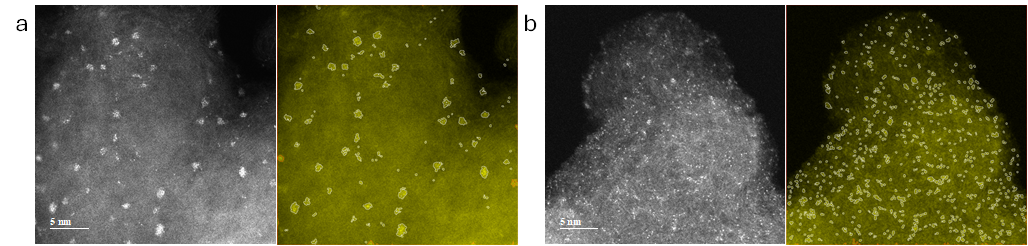


**Figure S43** | AC-STEM image and software image tracking of trimetallic NiPdPt SLMC and 3D cluster on Vulcan XC 72R without argon irradiation (a) and with argon irradiation (b).

**Table S16:** Summary information extracted by the STEM images quantitative analysis using the bespoke python software for the multimetallic single-layer metal cluster alloy.

| Metals | Ar^+^ | Number of SLMC | 3D Clusters | % of metal atoms as SLMC |
| --- | --- | --- | --- | --- |
| NiPd | No | 40 | 23 | 30% |
| NiPd | Yes | 621 | 4 | 98% |
| NiPt | No | 97 | 45 | 26% |
| NiPt | Yes | 526 | 3 | 98% |
| PdPt | No | 111 | 37 | 23% |
| PdPt | Yes | 876 | 11 | 96% |
| NiPdPt | No | 130 | 19 | 40% |
| NiPdPt | Yes | 483 | 0 | 100% |

**Figure S44** | XPS spectra for the NiPdPt@C 35%. a – shows the survey spectrum highlighting the Ni, Pd and Pt regions and b, c and d shows the Ni 2p, Pd 2d and Pt 4f high-resolution regions, respectively.

**
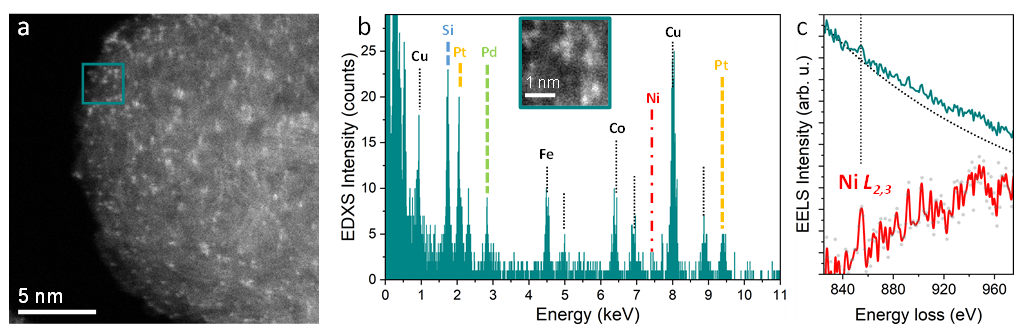
Figure S45** | a. AC-STEM image of the NiPdPt-C35%. Shows the region for EDX (b) and EELS (c). Supplementary Video 4 shows a movie across the analysis acquisition time, showing the dynamic of the single-atoms with the electron beam.

**
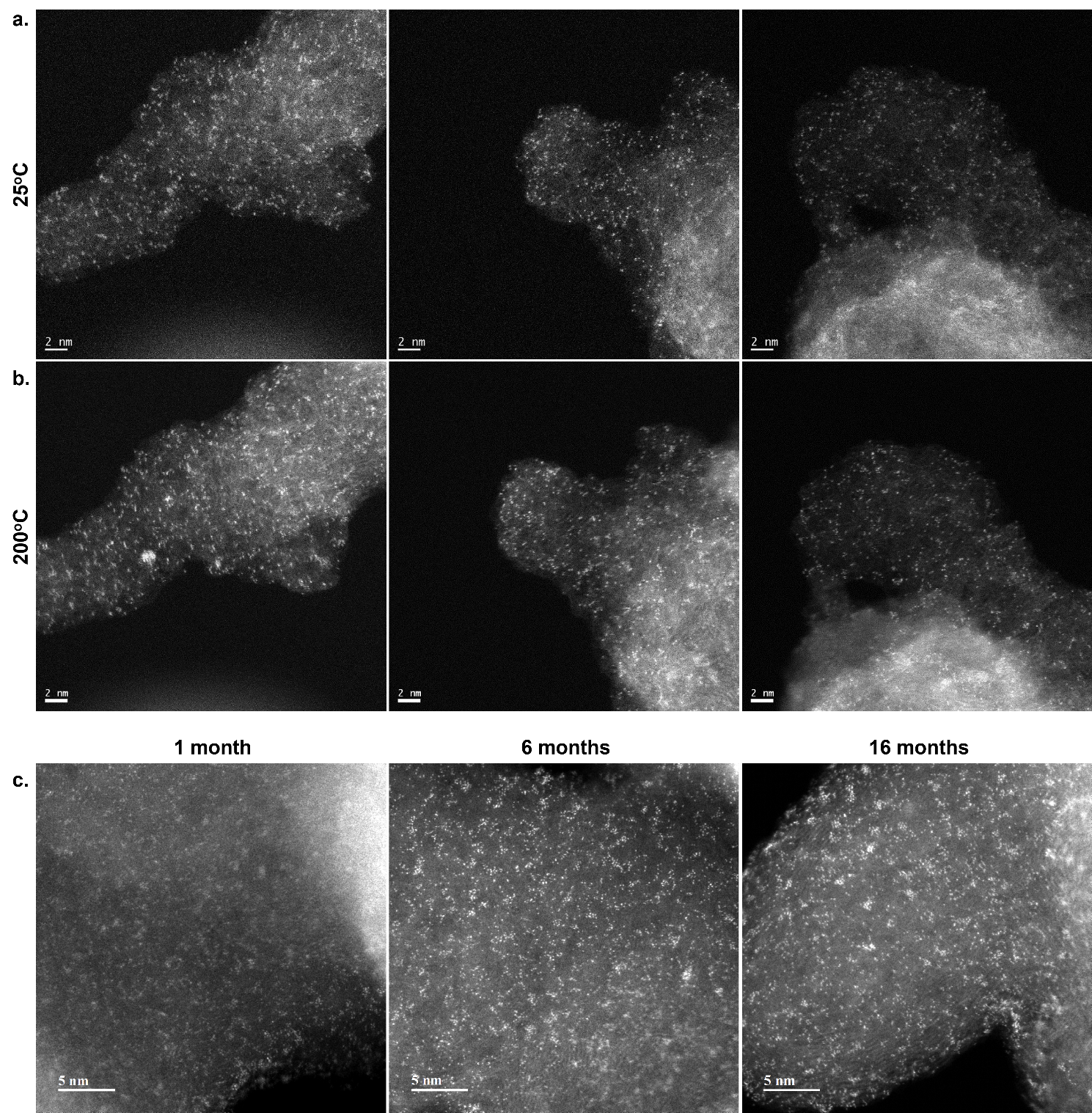
**

**Figure S46** | **Stability of NiPdPt-C35% SLMC at high temperature and over extended periods after production.** **a**, Identical-location AC-STEM images of SLMC on surface-engineered carbon as-prepared showing 3 different track regions. **b**, and AC-STEM images after thermal treatment at 200°C for 2 hours for the correspondent track region above panel **a**. **c**, AC-STEM images of NiPdPt SLMC on surface-engineered carbon after prolonged storage in the air following SLMC production.

*4.8 Electrochemistry experiments*


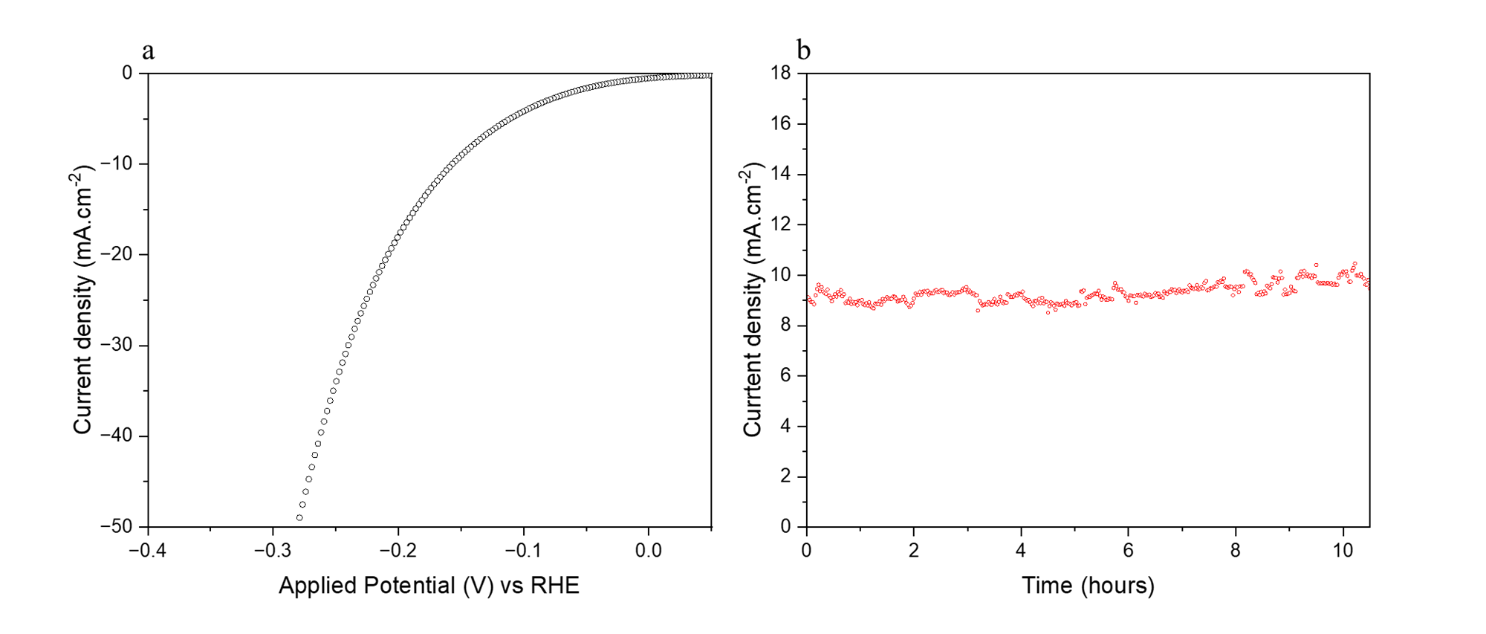


**Figure S47:** (a) Linear sweep voltammetry (LSV) of Pt–C35% measured in 0.5 M H₂SO₄, showing a hydrogen evolution onset potential of 0.16 V vs RHE at a current density of 10 mA cm⁻². (b) Chronoamperometric stability test of Pt–C35% at a fixed potential of 0.16 V vs RHE, demonstrating stable current density over 10 hours in 0.5 M H₂SO₄.

*4.9 Literature summary of methods for defect generation*

**Table S17:** Summary of methods of controlled defect formation in carbon lattice.

| **Method** | **Example** | **Reference** |
| --- | --- | --- |
| Ion bombardment | Ga^+^ focused ion beam (FIB) | *Science* 2014, **344**, 289 |
|  | He^+^ ion bombardment | *Nano Lett.* 2021, **21**, 2183 |
|  | Ga^+^ ion bombardment | *ACS Nano* 2017, **11**, 5726 |
| Thermal gas etching | O_2_ etching at 300 ^o^C | *ACS Appl. Mater. Interfaces* 2020,**12**, 36468 |
|  | O_3_ 25-100 ^o^C | *Nat. Commun.* 2018, **9**, 2632 |
| Wet chemical etching | KMnO_4_ in acidic solution | *Nano Lett.* 2014, **14**, 1234  *Nano Lett.* 2015, **15**, 3254 |
| Plasma | O_2_ and Ar plasma | *Adv. Funct. Mater.* 2020, **30**, 2003979  *Energy Environ. Sci.* 2019, **12**, 3305  *Sci. Adv.* 2019, **5**, eaav1851  *Nano Lett. 2021, 21, 12, 5179* |
| Electron beam bombardment | Etching by the 10 keV electron beam and surface adsorbed H_2_O | *Nature Commun*., 2021, **12**, 7170 |
|  | Direct momentum transfer from the 80 keV electron beam | *Nature Commun*., 2012, **3**, 1144 |
| Defects during growth | Chemical vapour deposition (CVD) | *Nat. Commun.* 2018, **9**, 2632  *Carbon* 2019, **153**, 4 |

**References**

[1] A. Mezzi, S. Kaciulis, *Surface and Interface Analysis* **2010**, 42, 1082.

[2] D. J. Morgan, *Surface Science Spectra* **2017**, 24, 024003.

[3] a)A. C. Ferrari, J. Robertson, *Physical Review B* **2000**, 61, 14095; b)M. Pawlyta, J. N. Rouzaud, S. Duber, *Carbon* **2015**, 84, 479.

[4] M. C. Biesinger, L. W. M. Lau, A. R. Gerson, R. S. C. Smart, *Applied Surface Science* **2010**, 257, 887.

[5] M. C. Biesinger, B. P. Payne, A. P. Grosvenor, L. W. M. Lau, A. R. Gerson, R. S. Smart, *Applied Surface Science* **2011**, 257, 2717.

[6] A. P. Grosvenor, B. A. Kobe, M. C. Biesinger, N. S. McIntyre, *Surface and Interface Analysis* **2004**, 36, 1564.

[7] A. P. Grosvenor, M. C. Biesinger, R. S. Smart, N. S. McIntyre, *Surface Science* **2006**, 600, 1771.

[8] M. C. Biesinger, *Surface and Interface Analysis* **2017**, 49, 1325.

[9] D. Barreca, G. A. Battiston, R. Gerbasi, E. Tondello, P. Zanella, *Surface Science Spectra* **2000**, 7, 303.

[10] Z. Weibin, W. Weidong, W. Xueming, C. Xinlu, Y. Dawei, S. Changle, P. Liping, W. Yuying, B. Li, *Surface and Interface Analysis* **2013**, 45, 1206.

[11] J. Baltrusaitis, B. Mendoza-Sanchez, V. Fernandez, R. Veenstra, N. Dukstiene, A. Roberts, N. Fairley, *Applied Surface Science* **2015**, 326, 151.

[12] D. J. Morgan, *Surface and Interface Analysis* **2015**, 47, 1072.

[13] R. J. Lewis, M. Koy, M. Macino, M. Das, J. H. Carter, D. J. Morgan, T. E. Davies, J. B. Ernst, S. J. Freakley, F. Glorius, G. J. Hutchings, *J Am Chem Soc* **2022**, 144, 15431.

[14] A. M. Ferraria, A. P. Carapeto, A. M. B. do Rego, *Vacuum* **2012**, 86, 1988.

[15] J. Wang, J. Fan, W. Wang, X. Zhang, L. Feng, X. Zhai, X. Shen, Z. Li, *RSC Adv* **2023**, 13, 10433.

[16] T. C. Tien, L. C. Lin, L. S. Lee, C. J. Hwang, S. Maikap, Y. M. Shulga, *Journal of Materials Science-Materials in Electronics* **2010**, 21, 475.

[17] R. A. McLellan, A. Dutta, C. Zhou, Y. Jia, C. Weiland, X. Gui, A. P. M. Place, K. D. Crowley, X. H. Le, T. Madhavan, Y. Gang, L. Baker, A. R. Head, I. Waluyo, R. Li, K. Kisslinger, A. Hunt, I. Jarrige, S. A. Lyon, A. M. Barbour, R. J. Cava, A. A. Houck, S. L. Hulbert, M. Liu, A. L. Walter, N. P. de Leon, *Adv Sci (Weinh)* **2023**, 10, e2300921.

[18] F. Y. Xie, L. Gong, X. Liu, Y. T. Tao, W. H. Zhang, S. H. Chen, H. Meng, J. Chen, *Journal of Electron Spectroscopy and Related Phenomena* **2012**, 185, 112.

[19] S. Iqbal, M. L. Shozi, J. Morgan, *Surface and Interface Analysis* **2017**, 49, 223.

[20] Q. Liu, Z. L. Zhang, *Catalysis Science & Technology* **2019**, 9, 4821.

[21] R. Radnik, C. Mohr, P. Claus, *Physical Chemistry Chemical Physics* **2003**, 5, 172.
